# Strategies to strengthen the resilience of primary health care in the COVID-19 pandemic: a scoping review

**DOI:** 10.1186/s12913-024-11278-4

**Published:** 2024-07-25

**Authors:** Ali Mohammad Mosadeghrad, Mahnaz Afshari, Parvaneh Isfahani, Farahnaz Ezzati, Mahdi Abbasi, Shahrzad Akhavan Farahani, Maryam Zahmatkesh, Leila Eslambolchi

**Affiliations:** 1 Health policy and management, Health Economics and Management Department, Tehran University of Medical Sciences, Tehran, Iran; 2 Health policy, School of Nursing and Midwifery, Saveh University of Medical Sciences, Saveh, Iran; 3 Health management, School of Public Health, Zabol University of Medical Sciences, Zabol, Iran; 4 Health services management, Health Economics and Management Department, Tehran University of Medical Sciences, Tehran, Iran; 5 Health Management, School of Business and Management, Royal Holloway University of London, London, UK

**Keywords:** Primary health care system, Resilience, Strengthening strategies, COVID-19, Crisis, Pandemic

## Abstract

**Background:**

Primary Health Care (PHC) systems are pivotal in delivering essential health services during crises, as demonstrated during the COVID-19 pandemic. With varied global strategies to reinforce PHC systems, this scoping review consolidates these efforts, identifying and categorizing key resilience-building strategies.

**Methods:**

Adopting Arksey and O'Malley's scoping review framework, this study synthesized literature across five databases and Google Scholar, encompassing studies up to December 31st, 2022. We focused on English and Persian studies that addressed interventions to strengthen PHC amidst COVID-19. Data were analyzed through thematic framework analysis employing MAXQDA 10 software.

**Results:**

Our review encapsulated 167 studies from 48 countries, revealing 194 interventions to strengthen PHC resilience, categorized into governance and leadership, financing, workforce, infrastructures, information systems, and service delivery. Notable strategies included telemedicine, workforce training, psychological support, and enhanced health information systems. The diversity of the interventions reflects a robust global response, emphasizing the adaptability of strategies across different health systems.

**Conclusions:**

The study underscored the need for well-resourced, managed, and adaptable PHC systems, capable of maintaining continuity in health services during emergencies. The identified interventions suggested a roadmap for integrating resilience into PHC, essential for global health security. This collective knowledge offered a strategic framework to enhance PHC systems' readiness for future health challenges, contributing to the overall sustainability and effectiveness of global health systems.

## Background

The health system is a complex network that encompasses individuals, groups, and organizations engaged in policymaking, financing, resource generation, and service provision. These efforts collectively aim to safeguard and enhance people health, meet their expectations, and provide financial protection [1]. The World Health Organization's (WHO) framework outlines six foundational building blocks for a robust health system: governance and leadership, financing, workforce, infrastructure along with technologies and medicine, information systems, and service delivery. Strengthening these elements is essential for health systems to realize their objectives of advancing and preserving public health [2].

Effective governance in health systems encompasses the organization of structures, processes, and authority, ensuring resource stewardship and aligning stakeholders’ behaviors with health goals [3]. Financial mechanisms are designed to provide health services without imposing financial hardship, achieved through strategic fund collection, management and allocation [4, 5]. An equitable, competent, and well-distributed health workforce is crucial in delivering healthcare services and fulfilling health system objectives [2]. Access to vital medical supplies, technologies, and medicines is a cornerstone of effective health services, while health information systems play a pivotal role in generating, processing, and utilizing health data, informing policy decisions [2, 5]. Collectively, these components interact to offer quality health services that are safe, effective, timely, affordable, and patient-centered [2]

The WHO, at the 1978 Alma-Ata conference, introduced primary health care (PHC) as the fundamental strategy to attain global health equity [6]. Subsequent declarations, such as the one in Astana in 2018, have reaffirmed the pivotal role of PHC in delivering high-quality health care for all [7]. PHC represents the first level of contact within the health system, offering comprehensive, accessible, community-based care that is culturally sensitive and supported by appropriate technology [8]. Essential care through PHC encompasses health education, proper nutrition, access to clean water and sanitation, maternal and child healthcare, immunizations, treatment of common diseases, and the provision of essential drugs [6]. PHC aims to provide protective, preventive, curative, and rehabilitative services that are as close to the community as possible [9].

Global health systems, however, have faced significant disruptions from various shocks and crises [10], with the COVID-19 pandemic being a recent and profound example. The pandemic has stressed health systems worldwide, infecting over 775 million and claiming more than 7.04 million lives as of April 13th, 2024 [11]. Despite the pandemic highlighting the critical role of hospitals and intensive care, it also revealed the limitations of specialized medicine when not complemented by a robust PHC system [12].

The pandemic brought to light the vulnerabilities of PHC systems, noting a significant decrease in the use of primary care for non-emergency conditions. Routine health services, including immunizations, prenatal care, and chronic disease management, were severely impacted [13]. The challenges—quarantine restrictions, fears of infection, staffing and resource shortages, suspended non-emergency services, and financial barriers—reduced essential service utilization [14]. This led to an avoidance of healthcare, further exacerbating health inequalities and emphasizing the need for more resilient PHC systems [15–17].

Resilient PHC systems are designed to predict, prevent, prepare, absorb, adapt, and transform when facing crises, ensuring the continuity of routine health services [18]. Investing in the development of such systems can not only enhance crisis response but also foster post-crisis transformation and improvement. This study focuses on identifying global interventions and strategies to cultivate resilient PHC systems, aiding policymakers and managers in making informed decisions in times of crisis.

## Methods

In 2023, we conducted a scoping review to collect and synthesize evidence from a broad spectrum of studies addressing the COVID-19 pandemic. A scoping review allows for the assessment of literature's volume, nature, and comprehensiveness, and is uniquely inclusive of both peer-reviewed articles and gray literature—such as reports, white papers, and policy documents. Unlike systematic reviews, it typically does not require a quality assessment of the included literature, making it well-suited for rapidly gathering a wide scope of evidence [19]. Our goal was to uncover the breadth of solutions aimed at bolstering the resilience of the PHC system throughout the COVID-19 crisis. The outcomes of this review are intended to inform the development of a model that ensures the PHC system's ability to continue delivering not just emergency services but also essential care during times of crisis.

We employed Arksey and O'Malley's methodological framework, which consists of six steps: formulating the research question, identifying relevant studies, selecting the pertinent studies, extracting data, synthesizing and reporting the findings, and, where applicable, consulting with stakeholders to inform and validate the results [20]. This comprehensive approach is designed to capture a wide range of interventions and strategies, with the ultimate aim of crafting a robust PHC system that can withstand the pressures of a global health emergency

### Stage 1: identifying the research question

Our scoping review was guided by the central question: "Which strategies and interventions have been implemented to enhance the resilience of primary healthcare systems in response to the COVID-19 pandemic?" This question aimed to capture a comprehensive array of responses to understand the full scope of resilience-building activities within PHC systems.

### Stage 2: identifying relevant studies

To ensure a thorough review, we conducted systematic searches across multiple databases, specifically targeting literature up to December 31st, 2022. The databases included PubMed, Web of Science, Scopus, Magiran, and SID. We also leveraged the expansive reach of Google Scholar. Our search strategy incorporated a bilingual approach, utilizing both English and Persian keywords that encompassed "PHC," "resilience," "strategies," and "policies," along with the logical operators AND/OR to refine the search. Additionally, we employed Medical Subject Headings (MeSH) terms to enhance the precision of our search. The results were meticulously organized and managed using the Endnote X8 citation manager, facilitating the systematic selection and review of pertinent literature.

### Stage 3: selecting studies

In the third stage, we meticulously vetted our search results to exclude duplicate entries by comparing bibliographic details such as titles, authors, publication dates, and journal names. This task was performed independently by two of our authors, LE and MA, who rigorously screened titles and abstracts. Discrepancies encountered during this process were brought to the attention of a third author, AMM, for resolution through consensus.

Subsequently, full-text articles were evaluated by four team members—LE, MA, PI, and SHZ—to ascertain their relevance to our research question. The selection hinged on identifying articles that discussed strategies aimed at bolstering the resilience of PHC systems amidst the COVID-19 pandemic Table 1.

**Table 1:** Search strategy in databases

**Databases**	**Search strategy**	**Initial search results**
PubMed	("Primary Health Care"[MeSH Terms] OR "Primary Health Care"[Title/Abstract] OR "Primary Healthcare"[Title/Abstract] OR "care primary health"[Title/Abstract] OR "Primary Care"[Title/Abstract] OR "care primary health"[Title/Abstract] OR "health care primary"[Title/Abstract] OR "Primary Healthcare"[Title/Abstract] OR "healthcare primary"[Title/Abstract] OR "Primary Care"[Title/Abstract] OR "care primary"[Title/Abstract]) AND ("COVID-19"[MeSH Terms] OR "SARS-CoV-2"[MeSH Terms] OR "COVID-19"[Title/Abstract] OR "COVID19"[Title/Abstract] OR "SARS-CoV-2"[Title/Abstract]) AND ("resilience"[Title/Abstract] OR "resiliences"[Title/Abstract] OR "resiliencies"[Title/Abstract] OR "resiliency"[Title/Abstract] OR "resilient"[Title/Abstract] OR "resilients"[Title/Abstract] OR "solutions"[MeSH Terms] OR "solutions"[Title/Abstract] OR "solution"[Title/Abstract] OR "strategie"[Title/Abstract] OR "strategies"[Title/Abstract] OR "strategy"[Title/Abstract] OR "strategy's"[Title/Abstract] OR "strengthen"[Title/Abstract] OR "strengthened"[Title/Abstract] OR "strengthening"[Title/Abstract] OR "strengthens"[Title/Abstract]) AND (english[Filter] OR persian[Filter])	1022
Scopus	ALL (COVID-19 OR coronaviru ) AND ALL ( "primary health care" OR "primary healthcare" OR "primary health-care" OR "primary care" ) AND ALL ( resilience OR strategies OR solution ) AND ( LIMIT-TO ( PUBSTAGE , "final" ) ) AND ( LIMIT-TO ( OA , "all" ) ) AND ( LIMIT-TO ( LANGUAGE , "English" ) OR LIMIT-TO ( LANGUAGE , "Persian" ) )	16489 [2000 results have been reviewed]
Web of Science	((ALL=( COVID-19 OR coronavirus)) AND ALL=(“primary health care” OR “primary healthcare” OR “primary health-care” OR “primary care” )) AND ALL=(resilience OR strategies OR solution) Refined By: Languages: English. Open Access: All Open Access	1102
Magiran	COVID-19 AND primary health care AND strategies	28
SID	COVID-19 AND primary health care AND resilience	13
Google scholar	Primary Health Care + resilience + COVID-19	345000 [150 results have been reviewed]
Total		4315

We have articulated the specific inclusion and exclusion criteria that guided our selection process in Table 2, ensuring transparency and replicability of our review methodology

**Table 2:** Inclusion and exclusion criteria for the scoping review

**Theme**	**Inclusion criteria**	**Exclusion criteria**
Population (Countries)	All countries	-
Concept	Interventions or strategies to strengthen PHC system in the COVID-19 pandemic.	Interventions or strategies to strengthen health systems in other settings or other emergencies such as natural or man-made disasters.
Context	PHC systems and during COVID-19 pandemic	Other health settings like hospital or outpatient clinics or in times rather than pandemic time
**Additional Filters**		
Type of Studies	Articles, documents, and reports	Thesis and book chapters
Language	English and Persian	Other languages
Date of Publication	Studies published the up to the search date( Dec 31st, 2022)	-

### Stage 4: charting the data

Data extraction was conducted by a team of six researchers (LE, MA, PI, MA, FE, and SHZ), utilizing a structured data extraction form. For each selected study, we collated details including the article title, the first author’s name, the year of publication, the country where the study was conducted, the employed research methodology, the sample size, the type of document, and the PHC strengthening strategies described.

In pursuit of maintaining rigorous credibility in our study, we adopted a dual-review process. Each article was independently reviewed by pairs of researchers to mitigate bias and ensure a thorough analysis. Discrepancies between reviewers were addressed through discussion to reach consensus. In instances where consensus could not be reached, the matter was escalated to a third, neutral reviewer. Additionally, to guarantee thoroughness, either LE or MA conducted a final review of the complete data extraction for each study.

### Stage 5: collating, summarizing and reporting the results

In this stage, authors LE, MZ, and MA worked independently to synthesize the data derived from the selected studies. Differences in interpretation were collaboratively discussed until a consensus was reached, with AMM providing arbitration where required.

We employed a framework thematic analysis, underpinned by the WHO's health system building blocks model, to structure our findings. This model categorizes health system components into six foundational elements: governance and leadership; health financing; health workforce; medical products, vaccines, and technologies; health information systems; and service delivery [2]. Using MAXQDA 10 software, we coded the identified PHC strengthening strategies within these six thematic areas.

## Results

### Summary of search results and study selection

**Fig. 1:**
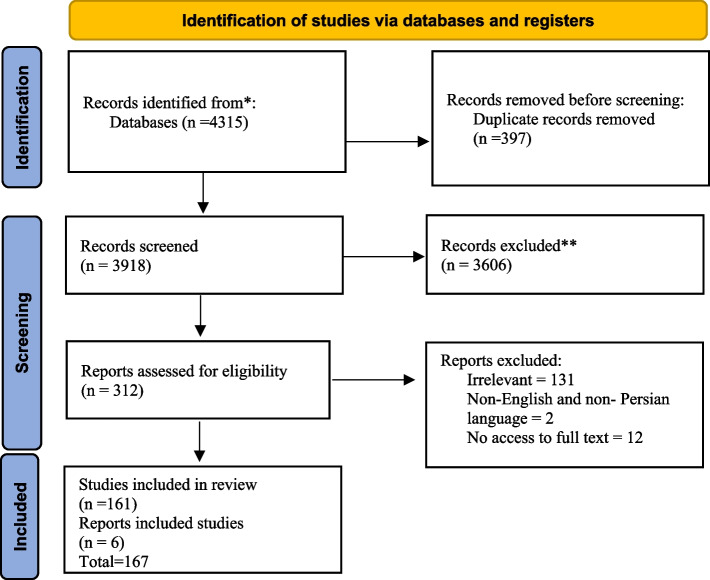
PRISMA Flowchart of search process and results

In total, 4315 articles were found by initial search. After removing 397 duplicates, 3918 titles and abstracts were screened and 3606 irrelevant ones were deleted. Finally, 167 articles of 312 reviewed full texts were included in data synthesis (Fig. 1). Main characteristics of included studies are presented in Appendix 1.

### Characteristics of studies

**Fig. 2:**
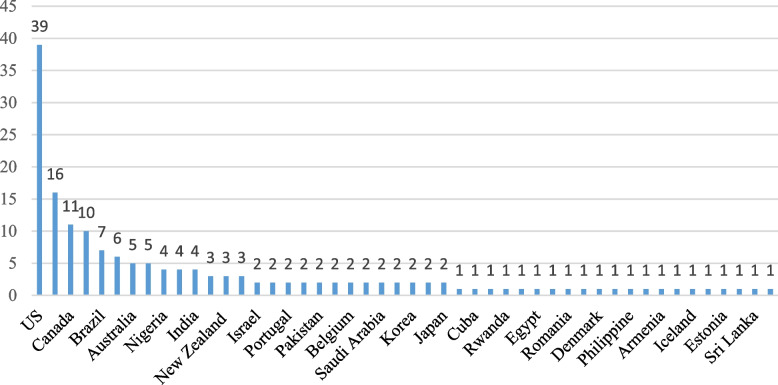
Distribution of reviewed studies by country

These studies were published in 2020 (18.6%), 2021 (36.5%) and 2022 (44.9%). They were conducted in 48 countries, mostly in the US (39 studies), the UK (16 studies), Canada (11 studies), Iran (10 studies) and Brazil (7 studies) as shown in Fig. 2.

**Fig. 3:**
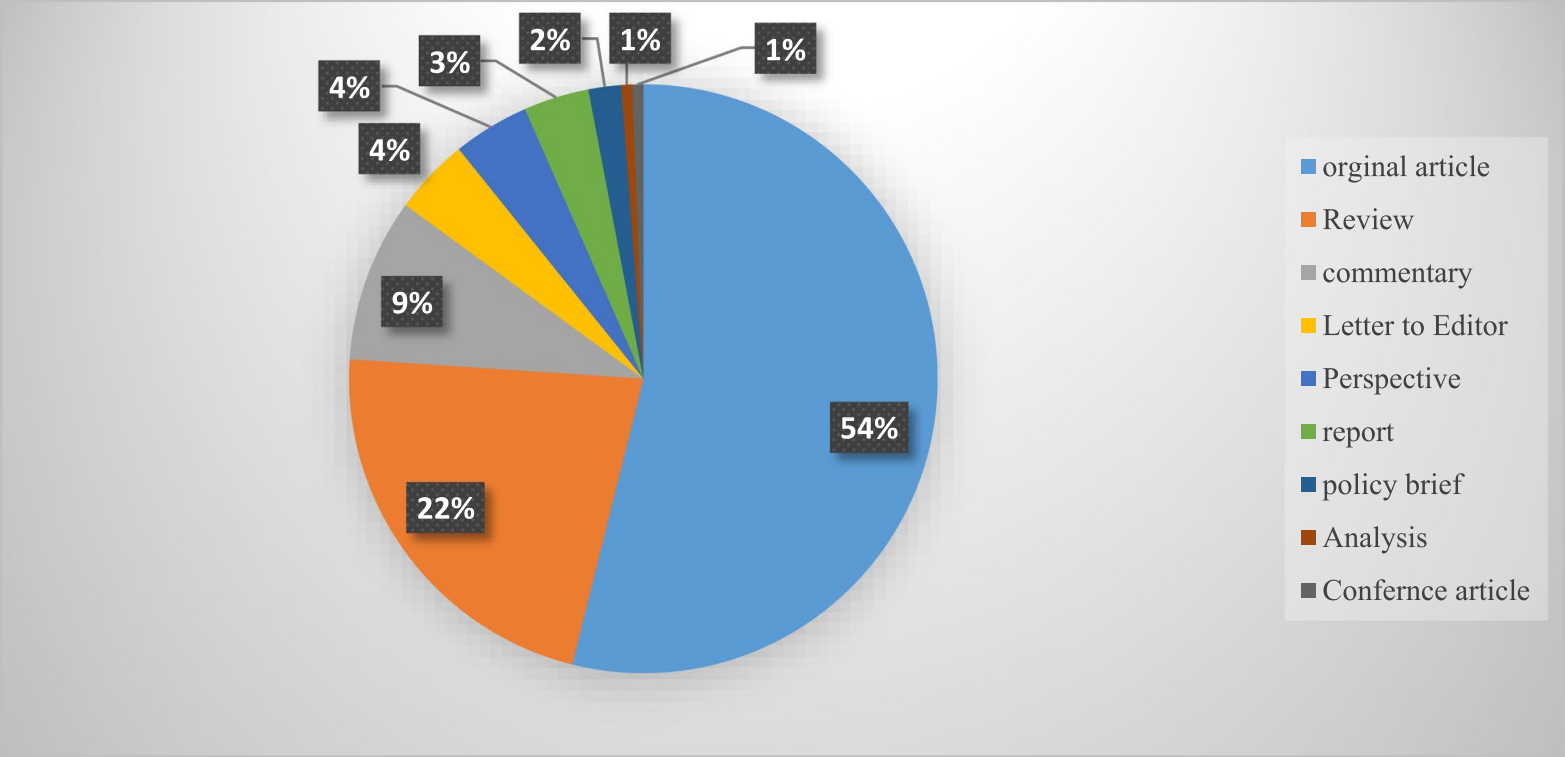
An overview of the publication types

Although the majority of the reviewed publications were original articles (55.1 %) and review papers (21 %), other types of documents such as reports, policy briefs, analysis, etc., were also included in this review (Fig. 3).

### Strengthening interventions to build a resilient PHC system

In total, 194 interventions were identified for strengthening the resilience of PHC systems to respond to the COVID-19 pandemic. They were grouped into six themes of PHC governance and leadership (46 interventions), PHC financing (21 interventions), PHC workforce (37 interventions), PHC infrastructures, equipment, medicines and vaccines (30 interventions), PHC information system (21 interventions) and PHC service delivery (39 interventions). These strategies are shown in Table 3.

**Table 3:** Interventions to strengthen the resilience of PHC systems to respond to COVID-19 pandemic

***PHC Governance and leadership***	
*** Organizational structure (6)***	Developing an integrated PHC system [21, 22]; Creating structural changes in PHC system as needed [23, 24]; Establishing a connection between public health and primary health care [25–28]; Appointing the national committee to monitor the performance of the PHC response [29]; Forming the Covid-19 research group [30, 31]; and creating a center to monitor the quality of vaccines [32].
*** Law & regulations (5)***	Creating legal frameworks for the right of people at risk to access basic health services [33, 34]; Initiating legislation for the integration of telehealth into existing practice models [35–38]; Passing the law on rights of telemedicine users and providers [35, 39]; Establishing laws to support the families of health service providers [40]; and Initiating legislation for full compensation of the cost of SARS-CoV-2 diagnostic testing and paramedics [35].
*** Policies & plans (13)***	Collaborative planning and policy making [41–43]; Evidence based planning and policy making [44–47]; Developing the national pandemic preparedness program [46, 48, 49]; Integrating the PHC into disaster and emergency management policies [50]; Planning the essential services maintenance [51]; Prioritizing pandemic response efforts for vulnerable and sensitive populations [17, 38, 52–58]; Planning the **i**ncident response [50]; Developing the post-pandemic recovery plan [43]; Planning for sharing resources in a district [43]; Linking strategic plans for immunization to national health plans [52]; Enforcing family medicine program [56]; Adapting comprehensive and multi-sectoral strategies [59]; and Using the capacity of the private sector to increase the coverage of health services [45, 59].
*** Communications & collaborations (6)***	Strengthening communication systems and flows [29, 34, 43, 49, 54, 60–63]; Strengthening collaboration between the national pandemic response team and the PHC authorities [28, 29, 47, 57, 63–67]; Strengthening international collaborations [54, 63]; Improving international communications and information sharing [68]; Enforcing public-private collaborative governance [59, 69, 70]; and Collaborating with national and international educational and research networks [56, 68, 71].
*** Community participation (8)***	Developing community engagement and awareness protocols [54, 72], Creating a platform for expressing community needs [73]; Increasing community health literacy [45, 49, 74–77]; Educating patients and professionals about the virtual care process [78, 79]; Encouraging the participation of the health system experts in social media [68]; Empowering and informing the public through different media [21, 49, 53, 80, 81]; Establishing awareness campaigns [32, 41, 46, 57, 82–85] and Attracting the participation of leaders, donors and non-governmental organizations [21, 25, 32, 40, 86].
*** Effective leadership (2)***	Dynamic and responsive leadership [45], Community leadership development [28, 58].
*** Monitoring & evaluation (6)***	Establishing effective and well-integrated surveillance systems [54, 61, 87]; Establishing monitoring and evaluation system [21, 28, 31, 32, 46–49, 54, 61, 63, 88–91]; Establishing quality control system [47, 85, 92]; Administering quarterly districts-level stress tests [43]; Performing infrastructure and technology safety assessments [50]; and Accrediting the integration of tele-health into existing health system [36].
***PHC Financing***	
*** Revenue raising (9)***	Capacity building of states in health financing [43, 56]; Allocating sufficient funds [54, 67, 70, 85, 93–95]; Assuring sustainable funding [45, 68, 81]; Allocating independent funds for epidemics and pandemics management [45]; Allocating adequate financial resources for PHC [27, 45, 52, 56, 61, 96]; Providing mechanisms to rapidly mobilize funds [43, 52, 54]; Using the financial reserve [45]; National and international borrowing [25, 45]; and Funding for separate areas, workforce and telehealth [65].
*** Pooling of funds (7)***	Reinforcing the health insurance scheme [97]; Encouraging private insurance companies’ participation in financing care during the pandemic [48, 52]; Launching state-specific free medicines and diagnostics schemes [50]; Providing free medical care and treatment [82, 98]; Regional sharing and efficient use of resources [99]; Increasing financial support plans for patients [48, 87]; and Making partnership with phone companies to waive data charges for telemedicine-related services [100].
*** Purchasing of health services (5)***	Using strategic purchasing [52]; Increasing purchasing flexibility [54, 101]; Reimbursing virtual care and telemedicine services [65, 78, 102, 103, 103, 104]; Optimizing the balance of remote and in-person care payment for older adults [105] and Purchasing basic and para-clinical services from the private sector [21].
***PHC Workforce***	
*** Recruitment (8)***	Proper allocation and distribution of manpower [29, 31, 44, 56, 84, 87, 106]; Building capacity for rapid mobilization of health workers [29, 43, 54, 107, 108]; Ensuring an adequate number of staff and hiring additional ones [17, 28, 29, 45, 63, 81, 85, 99, 109]; Having clear and transparent workflow [34]; Determining the roles and responsibilities of all employees with focus on paramedics and local workers [45, 63]; Recruiting technological support staff [102]; Encouraging students to participate in providing services [41, 73, 109]; and Recruiting and training volunteers [29, 40, 54].
*** Training & development (6)***	Developing new training programs [28, 43, 48, 72, 78, 81, 95, 100, 103, 105, 110–115]; Continuous education and training [31, 40, 47, 54, 56, 63, 72, 76, 80, 84, 85, 87, 88, 91, 92, 101, 105, 108, 116–120]; Educational collaboration with Virtual University [21]; Upgrading academic curricula [121]; Increasing the student admission capacity [121, 122]; and Providing intensive nursing training courses [121].
*** Teamwork (7)***	Strengthening the existing network of community health workers [63, 65, 97, 123]; Forming multi-disciplinary primary care teams [17, 24, 27]; Forming patient-centered teams [104, 124]; Forming rapid response public health teams [45, 95]; Forming COVID-19 (Emergency and risk) management teams [29]; Appointing facility-based monitoring team [29]; and Effective team communication and collaboration [43, 81, 101, 108, 125].
*** Protection (12)***	Developing physical safety plan [85, 92, 122]; Periodic controlling of the health status of the employees and large-scale screening [44, 48]; Providing suitable and well-ventilated workspace [45]; Promoting self-care [45, 126, 127]; Applying personal resilience-building interventions [43, 45, 110, 114, 119, 128–131]; Providing psychological support [45, 54, 58, 85, 87, 89, 96, 107, 109, 116, 119, 126, 128, 131–133]; Reducing the job stress [45, 134]; Ensuring access to testing for all staff [112, 135]; Using relaxation mechanisms [133]; Reducing reporting requirements [29]; Vaccinating the staff [63]; and Modifying the work schedule [29, 107, 126, 136].
*** Performance appraisal (1)***	Continuous evaluation of employees’ performance [49].
*** Compensation and reward (3)***	Designing an efficient incentive system [21, 27, 63, 136]; Using proper initiatives to motivate staffs [40, 46]; and Providing financial support [54, 109].
***PHC Infrastructures, medical products and equipment***	
*** Healthcare facilities (8)***	Infection prevention and control measures [137]; Reducing structural and physical obstacles to access facilities [52, 54, 138]; Setting up vaccination centers in different places [32, 139]; Setting up point of care testing laboratory [42, 137]; Creating molecular diagnostic laboratories in provinces [98]; Providing centralized access centers for unattached patients [140]; Launching COVID-19 dedicated primary healthcare clinics [34, 51]; and Providing proper physical infrastructure for safe long-term storage of drugs and buffer stock [115].
*** Medicine & Diagnostic kits (5)***	Storing and redistributing basic medicines [21]; Providing sufficient supplies of medicine and diagnostic kits [43, 45, 61, 63, 87, 88, 141, 142]; Preparing inventoried list of medicines, supplies, and devices (or lab equipment) [50]; Creating a platform for the exchange of unused medical supplies between people [73]; and Mobilizing the drug delivery systems in remote areas with public-private partnership [34].
*** Vaccines (4)***	Using authentic vaccines [32]; Improving the quality of vaccines [32]; Receiving international vaccine approvals [32]; and Facilitating the import of vaccines [32].
*** Medical equipment (5)***	Promoting domestic capacity for medical technology [87]; Providing ultrasound diagnostics devices [36]; Providing adequate protection equipment [40, 45, 47, 51, 61, 63, 88, 112, 116, 118, 143, 144]; Providing telehealth equipment and digital diagnostic devices [17, 36, 145]; and Providing mobile health tools [146].
*** Digital infrastructure (8)***	Developing fully functional telecommunications infrastructure with backup [27, 29, 34, 62, 67, 73, 81, 89, 102, 104, 113, 116, 147, 148]; Setting up free Wi-Fi hotspots [100], Developing centralized public online booking systems [140]; Launching virtual clinics [149]; Developing an online referral system [94]; Developing universal centralized electronic immunization records [51]; Creating a website to manage voluntary donations [86]; and Developing mobile health applications [80, 132, 149].
***PHC Information system***	
*** Information systems (11)***	Developing robust reliable health information system [45, 46, 49, 64, 87, 106, 150]; Developing an offline health management information system [115]; Developing electronic health records [21–23, 37, 106, 113]; Developing strong surveillance system [26, 47, 61, 63, 74, 95]; Universal centralized electronic immunization records [51]; Linking the existing data systems [21]; Creating management dashboards [23]; Mapping health facilities regularly [43]; Controlling big data and inventory [24, 56, 92, 95, 106, 111]; Establishing and coordinating early warning systems [38, 63]; and Using assistive intelligent technologies [68, 151].
*** Research (10)***	Participating in national and international research networks [68]; Applying projections and modeling [52, 71, 90]; Conducting research on good practices, new information, diagnostic methods, and treatment of COVID-19 [43, 44, 46, 68, 76, 152]; Piloting the implementation of plans to deal with the pandemic [63]; Mapping barriers to access essential services [43]; Determining socio-economic effects of quarantine [45, 101]; Conducting weekly telephone surveys [89]; Providing real-time feedback and guidance to inform policy for future pandemics management [43, 47]; Investing in science, technology, and innovation [22]; and Improving the sharing of individual and population data and knowledge [26, 54, 113].
***PHC delivery***	
*** Defining services (14)***	Defining integrated, alternative and flexible services [21, 111]; Revising services and classifying them into in-person and out-of-person care departments [23]; Defining clear essential health services package [43, 54, 116]; Enabling patient-centric care [104, 124]; Implementing integrated non-communicable diseases prevention and management program [93]; Linking immunization into national health plans [52]; Incorporating screening services in a basic health package [82]; Providing emergency referral and transport [34, 45, 49]; Providing nurse-led primary care [153]; Establishing a mobile clinics [154, 155]; Launching fever clinics [25]; Providing home- based health services [47, 96, 136, 138, 156, 157]; Arranging shared medical appointments to address several health problems of an individual [158]; and Designing specific and timely processes for laboratory tests [63].
*** Developing and Using guidelines (8)***	Using WHO PHC COVID-19 Guidance and checklists [29]; Non-physical examination guideline [60]; Antiviral treatment guideline [63]; Guideline for communicable and non- communicable disease management in pandemics [31, 135]; Mental health services guideline; Face to face care guideline [135]; Guidelines for the care of vulnerable groups [23] ; and Telehealth guidelines [38, 39, 89, 147].
*** In-person services (7)***	Finding, testing, tracing, and quarantining patients [24, 28, 45, 48, 54, 63, 65, 91, 94, 133, 135, 159]; Having effective COVID-19 vaccination [25, 45, 53, 83, 160]; Supporting people during quarantine or isolation [54, 65, 77, 82, 112, 159]; Administering rapid diagnostic tests [39, 69, 135]; Maintaining regular PHC services [26, 45, 54, 56, 61, 66, 68, 87, 161]; Providing mental health and social care [38, 74, 78, 120, 123, 140, 162]; and Providing health education services [78, 82, 138].
*** Telehealth services (10)***	Arranging non-attendance referrals [21, 92]; Remote monitoring and follow-up [17, 29, 62, 135, 138, 163, 164]; Providing telemedicine visits [17, 25, 29, 34, 36–39, 45, 47, 49, 62, 67, 69, 76, 81, 94, 99–101, 109, 112, 122, 137, 149, 158, 160, 165–172]; Providing teleconsultation [23, 39, 49, 89, 91, 103, 139, 141, 147, 157, 163, 173–176]; Offering tele-pharmacy services [177]; Conducting telephone verbal screening [23]; Doing robotic triage [145]; Launching COVID-19 and mental hotlines [80, 140, 149]; Offering mobile health services [105, 149, 170]; and Offering online health services [17, 21, 124, 138, 167].

## Discussion

This scoping review aimed to identify and categorize the range of interventions employed globally to strengthen the resilience of primary healthcare (PHC) systems in the face of the COVID-19 pandemic. Our comprehensive search yielded 194 distinct interventions across 48 countries, affirming the significant international efforts to sustain healthcare services during this unprecedented crisis. These interventions have been classified according to the WHO’s six building block model of health systems, providing a framework for analyzing their breadth and depth. This review complements and expands upon the findings from Pradhan et al., who identified 28 interventions specifically within low and middle-income countries, signaling the universality of the challenge and the myriad of innovative responses it has provoked globally [178].

The review highlights the critical role of governance and leadership in PHC resilience. Effective organizational structure changes, legal reforms, and policy development were crucial in creating adaptive healthcare systems capable of meeting the dynamic challenges posed by the pandemic. These findings resonate with the two strategies of effective leadership and coordination emphasized by Pradhan et al. (2023), and underscore the need for clear vision, evidence-based policy, and active community engagement in governance [178]. The COVID-19 pandemic posed significant challenges for PHC systems globally. A pivotal response to these challenges was the active involvement of key stakeholders in the decision-making process. This inclusivity spanned across the spectrum of general practitioners, health professionals, health managers, and patients. By engaging these vital contributors, it became possible to address their specific needs and to design and implement people-centered services effectively [41–43].

The development and implementation of collaborative, evidence-informed policies and national healthcare plans were imperative. Such strategies required robust leadership, bolstered by political commitment, to ensure that the necessary changes could be enacted swiftly and efficiently [41, 45]. Leaders within the health system were called upon to foster an environment of good governance. This entailed promoting increased participation from all sectors of the healthcare community, enhancing transparency in decision-making processes, and upholding the principles of legitimacy, accountability, and responsibility within the health system [10]. The collective aim was to create a more resilient, responsive, and equitable healthcare system in the face of the pandemic's demands.

In the wake of the COVID-19 pandemic, governments were compelled to implement new laws and regulations. These were designed to address a range of issues from professional accreditation and ethical concerns to supporting the families of healthcare workers. Additionally, these legal frameworks facilitated the integration of emerging services such as telemedicine into the healthcare system, ensuring that these services were regulated and standardized [38, 40, 61]. A key aspect of managing the pandemic was the establishment of effective and transparent communication systems for patients, public health authorities, and the healthcare system at large [60, 61]. To disseminate vital information regarding the pandemic, vaccination programs, and healthcare services, authorities leveraged various channels. Public media, local online platforms, and neighborhood networks were instrumental in keeping the public informed about the ongoing situation and available services [53, 60, 86]. For health professionals, digital communication tools such as emails and WhatsApp groups, as well as regular meetings, were utilized to distribute clinical guidelines, government directives, and to address any queries they might have had. This ensured that healthcare workers were kept up-to-date with the evolving landscape of the pandemic and could adapt their practices accordingly [60, 144].

Healthcare facilities function as complex socio-technical entities, combining multiple specialties and adapting to the ever-changing landscape of healthcare needs and environments [179]. To navigate this dynamic, policy makers must take into account an array of determinants—political, economic, social, and environmental—that influence health outcomes. Effective management of a health crisis necessitates robust collaboration across various sectors, including government bodies, public health organizations, primary healthcare systems, and hospitals. Such collaboration is not only pivotal during crisis management but also during the development of preparedness plans [63]. Within the health system, horizontal collaboration among departments and vertical collaboration between the Ministry of Health and other governmental departments are vital. These cooperative efforts are key to reinforce the resilience of the primary healthcare system. Moreover, a strong alliance between national pandemic response teams and primary healthcare authorities is essential to identifying and resolving issues within the PHC system [29]. On an international scale, collaborations and communications are integral to the procurement of essential medical supplies, such as medicines, equipment, and vaccines. These international partnerships are fundamental to ensuring that health systems remain equipped to face health emergencies [63].

To ensure the PHC system's preparedness and response capacity was at its best, regular and effective monitoring and evaluation programs were put in place. These included rigorous quarterly stress tests at the district level, which scrutinized the infrastructure and technology to pinpoint the system’s strengths and areas for improvement [43]. Furthermore, clinical audits were conducted to assess the structure, processes, and outcomes of healthcare programs, thereby enhancing the quality and effectiveness of the services provided [63]. These evaluation measures were crucial for maintaining a high standard of care and for adapting to the ever-evolving challenges faced by the PHC system.

Financial strategies played a critical role in enabling access to essential health services without imposing undue financial hardship. Various revenue-raising, pooling, and purchasing strategies were implemented to expand PHC financing during the pandemic, illustrating the multifaceted approach needed to sustain healthcare operations under strained circumstances [9, 19].

In response to the COVID-19 pandemic, the Indian government took decisive action to bolster the country's healthcare infrastructure. By enhancing the financial capacity of states, the government was able to inject more funds into the Primary Health Care (PHC) system. This influx of resources made it possible to introduce schemes providing free medications and diagnostic services [50]. The benefits of increased financial resources were also felt beyond India's borders, enabling the compensation of health services in various forms. In Greece, it facilitated the monitoring and treatment of COVID-19 through in-person, home-based, and remote health services provided by physicians in private practice. Similarly, in Iran, the financial boost supported the acquisition of basic and para-clinical services from the private sector [21, 65]. These measures reflect a broader international effort to adapt and sustain health services during a global health crisis.

The COVID-19 pandemic presented a formidable challenge to the PHC workforce worldwide. Healthcare workers were subjected to overwhelming workloads and faced significant threats to both their physical and mental well-being. To build resilience in the face of this crisis, a suite of interventions was implemented. These included recruitment strategies, training and development programs, enhanced teamwork, improved protective measures, comprehensive performance appraisals, and appropriate compensation mechanisms, as documented in Table 3. To address staffing needs within PHC centers, a range of professionals including general practitioners, nurses, community health workers, and technical staff were either newly employed or redeployed from other healthcare facilities [63]. Expert practitioners were positioned on the frontlines, providing both in-person services and telephone consultations, acting as gatekeepers in the health system [49, 63]. Support staff with technological expertise played a crucial role as well, assisting patients in navigating patient portals, utilizing new digital services, and conducting video visits [102]. Furthermore, the acute shortage of healthcare workers was mitigated by recruiting individuals who were retired, not currently practicing, or in training as students, as well as by enlisting volunteers. This strategy was key to bolstering the workforce and ensuring continuity of care during the pandemic [109].

During the pandemic, new training programs were developed to prepare healthcare staff for the evolving demands of their roles. These comprehensive courses covered a wide array of critical topics, including the correct use of personal protective equipment (PPE), the operation of ventilators, patient safety protocols, infection prevention, teamwork, problem-solving, self-care techniques, mental health support, strategies for managing stress, navigating and applying reliable web-based information, emergency response tactics, telemedicine, and direct care for COVID-19 patients [74, 95, 100, 108, 110, 112, 117].

Acknowledging the psychological and professional pressures faced by the primary healthcare workforce, health managers took active measures to safeguard both the physical and mental well-being of their employees during this challenging period [124]. Efforts to protect physical health included monitoring health status, ensuring vaccination against COVID-19, and providing adequate PPE [63, 72]. To address mental health, a variety of interventions were deployed to mitigate anxiety and related issues among frontline workers. In Egypt, for instance, healthcare workers benefited from psychotherapy services and adaptable work schedules to alleviate stress [126]. Singapore employed complementary strategies, such as yoga, meditation, and the encouragement of religious practices, to promote relaxation among staff [133]. In the United States, the Wellness Hub application was utilized as a tool for employees to enhance their mental health [132]. In addition to health and wellness initiatives, there were financial incentives aimed at motivating employees. Payment protocols were revised, and new incentives, including scholarship opportunities and career development programs, were introduced to foster job satisfaction and motivation among healthcare workers [63].

The resilience of PHC systems during the pandemic hinged on several key improvements. Enhancing health facilities, supplying medicines and diagnostic kits, distributing vaccines, providing medical equipment, and building robust digital infrastructure were all fundamental elements that contributed to the strength of PHC systems, as outlined in Table 3. Safe and accessible primary healthcare was facilitated through various means. Wheelchair routes were created for patients to ensure their mobility within healthcare facilities. , dedicated COVID-19 clinics were established, mass vaccination centers were opened to expedite immunization, and mobile screening stations were launched to extend testing capabilities [23, 33, 63, 140].

In Iran, the distribution and availability of basic medicines were managed in collaboration with the Food and Drug Organization, ensuring that essential medications reached those in need [89]. During the outbreak, personal protective equipment (PPE) was among the most critical supplies. Access to PPE was prioritized, particularly for vulnerable groups and healthcare workers, to provide a layer of safety against the virus [63]. Vaccines were made available at no cost, with governments taking active measures to monitor their safety and side effects, to enhance their quality, and to secure international approvals. Furthermore, effective communication strategies were employed to keep the public informed about vaccine-related developments [32, 83].

These comprehensive efforts underscored the commitment to maintaining a resilient PHC system in the face of a global health every individual in the community could access healthcare services. To facilitate this, free high-speed Wi-Fi hotspots were established, enabling patients to engage in video consultations and utilize a range of e-services without the barrier of internet costs crisis. Significant enhancements were made to the digital infrastructure. This expansion was critical in ensuring that [30, 54]. Complementing these measures, a variety of digital health tools were deployed to further modernize care delivery. Countries like Nigeria and Germany, for instance, saw the introduction of portable electrocardiograms and telemedical stethoscopes. These innovations allowed for more comprehensive remote assessments and diagnostics, helping to bridge the gap between traditional in-person consultations and the emerging needs for telemedicine [141, 180].

Throughout the COVID-19 pandemic, targeted interventions were implemented to bolster information systems and research efforts, as outlined in Table 3. Key among these was the advancement of a modern, secure public health information system to ensure access to health data was not only reliable and timely but also transparent and accurate [33, 45, 49]. The "Open Notes" initiative in the United States exemplified this effort, guaranteeing patient access to, and editorial control over, their health records [141]. Management strategies also promoted the "one-health" approach, facilitating the exchange of health data across various departments and sectors to enhance public health outcomes [10].

In addition to these information system upgrades, active patient surveillance and early warning systems were instituted in collaboration with public health agencies. These systems played a pivotal role in detecting outbreaks, providing precise reports on the incidents, characterizing the epidemiology of pathogens, tracking their spread, and evaluating the efficacy of control strategies. They were instrumental in pinpointing areas of concern, informing smart lockdowns, and improving contact tracing methods [33, 63, 72]. The reinforcement of these surveillance and warning systems had a profound impact on shaping and implementing a responsive strategy to the health crisis [10].

To further reinforce the response to the pandemic, enhancing primary healthcare (PHC) research capacity became crucial. This enabled healthcare professionals and policymakers to discern both facilitators and barriers within the system and to devise fitting strategies to address emerging challenges. To this end, formal advisory groups and multidisciplinary expert panels, which included specialists from epidemiology, clinical services, social care, sociology, policy-making, and management, were convened. These groups harnessed the best available evidence to inform decision-making processes [30]. Consequently, research units were established to carry out regular telephone surveys and to collect data on effective practices, as well as new diagnostic and therapeutic approaches [31, 89]. The valuable insights gained from these research endeavors were then disseminated through trusted channels to both the public and policymakers, ensuring informed decisions at all levels [36].

The COVID-19 pandemic acted as a catalyst for the swift integration of telemedicine into healthcare systems globally. This period saw healthcare providers leverage telecommunication technologies to offer an array of remote services, addressing medical needs such as consultations, diagnosis, monitoring, and prescriptions. This transition was instrumental in ensuring care continuity and mitigating infection risks for both patients and healthcare workers, highlighting an innovative evolution in healthcare delivery [170, 181].

Countries adapted to this new model of healthcare with varied applications: Armenia established telephone follow-ups and video consultations for remote patient care, while e-pharmacies and mobile health tools provided immediate access to medical information and services [29]. In France and the United States, tele-mental health services and online group support became a means to support healthy living during the pandemic [147, 158] . New Zealand introduced the Aroha chatbot, an initiative to assist with mental health management [139].

The implementation and effectiveness of these telehealth services were not limited by economic barriers, as underscored by Pradhan et al. (2023), who noted the key role of telemedicine in low and middle-income countries. These countries embraced the technology to maintain health service operations, proving its global applicability and utility [178]. The widespread adoption of telemedicine, therefore, represents a significant and perhaps lasting shift in healthcare practice, one that has redefined patient care in the face of a global health crisis and may continue to shape the future of healthcare delivery [170, 178, 181].

## Conclusion

The study highlighted PHC strengthening strategies in COVID-19 time . Notably, the adaptations and reforms spanned across governance, financing, workforce management, information system, infrastructural readiness, and service delivery enhancements. These interventions collectively contributed to the robustness of health systems against the sudden surge in demand and the multifaceted challenges imposed by the pandemic and resulted.

**Fig. 4:**
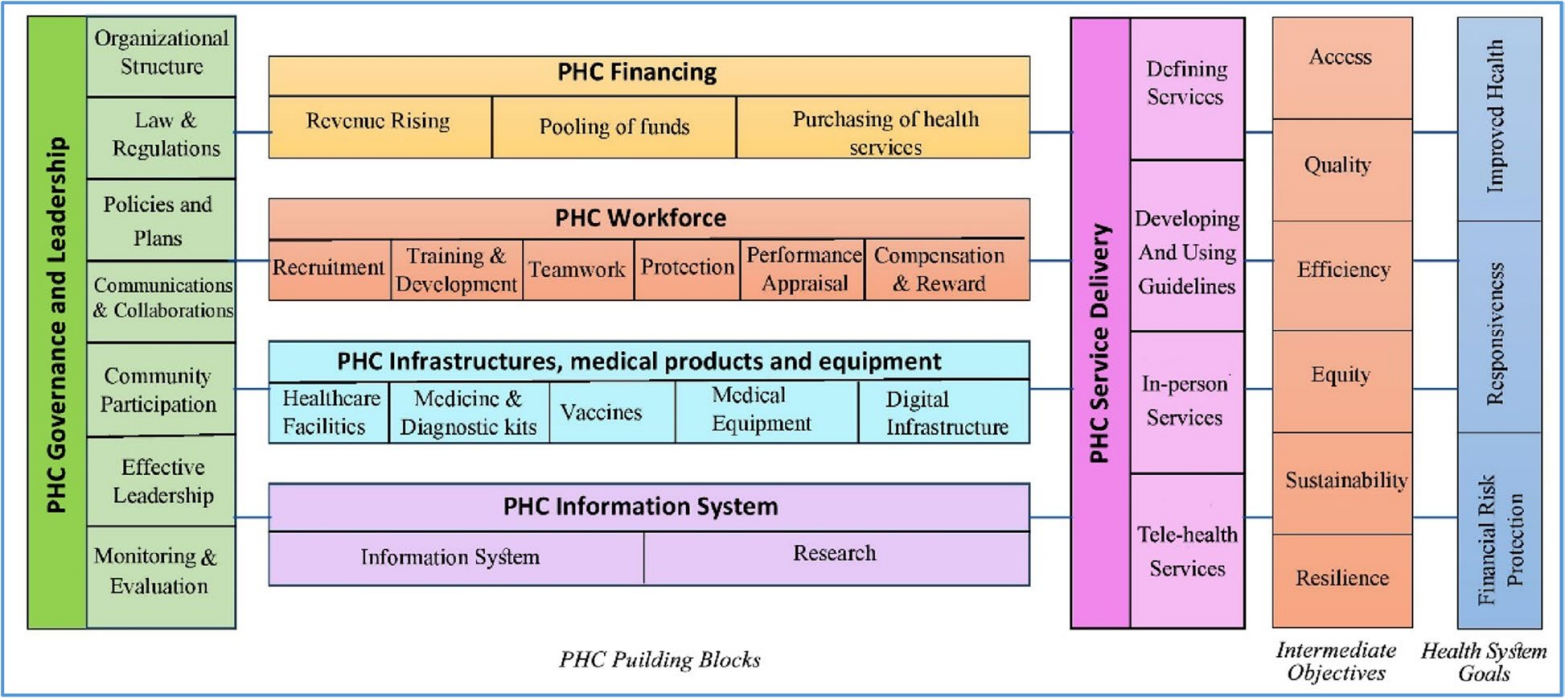
A model for strengthening the resilience of the primary health care system

Significantly, the findings have broader implications for health policy and system design worldwide. The pandemic has highlighted the critical need for resilient health systems that are capable of not only responding to health emergencies but also maintaining continuity in essential services. The strategies documented in this review serve as a template for countries to fortify their health systems by embedding resilience into their PHC frameworks (Fig. 4). Future health crises can be better managed by learning from these evidenced responses, which emphasize the necessity of integrated, well-supported, and dynamically adaptable health care structures.

Looking ahead, realist reviews could play a pivotal role in refining PHC resilience strategies. By understanding the context in which specific interventions succeed or fail, realist reviews can help policymakers and practitioners design more effective health system reforms, as echoed in the need for evidence-based planning in health system governance [9] ​​. These reviews offer a methodological advantage by focusing on the causality between interventions and outcomes, aligning with the importance of effective health system leadership and management [50, 182] ​​. They take into account the underlying mechanisms and contextual factors, thus providing a nuanced understanding that is crucial for tailoring interventions to meet local needs effectively [28, 86] ​​, ultimately leading to more sustainable health systems globally. This shift towards a more analytical and context-sensitive approach in evaluating health interventions, as supported by WHO's framework for action [2, 10] ​​, will be crucial for developing strategies that are not only effective in theory but also practical and sustainable in diverse real-world settings.

## Limitations and future research

In our comprehensive scoping review, we analyzed 167 articles out of a dataset of 4,315, classifying 194 interventions that build resilience in primary healthcare systems across the globe in response to pandemics like COVID-19. While the review's extensive search provides a sweeping overview of various strategies, it may not capture the full diversity of interventions across all regions and economies. Future research should focus on meta-analyses to evaluate the effectiveness of these interventions in greater detail and employ qualitative studies to delve into the specific challenges and successes, thus gaining a more nuanced understanding of the context. As the review includes articles only up to December 31, 2022, it may overlook more recent studies. Regular updates, a broader linguistic range, and the inclusion of a more diverse array of databases are recommended to maintain relevance and expand the breadth of literature, ultimately guiding more focused research that could significantly enhance the resilience of PHC systems worldwide.

## Data Availability

The datasets used and/or analyzed during the current study available from the corresponding author on reasonable request.

## Appendix 1

**Table 4:** Main characteristics of included studies

**No**	**First Author’s Name**	**Year of Publication**	**Country**	**Type**	**Method**	**Reference**
1	Bipin Adhikari	2022	UK	Review	Scoping review (narrative synthesis of literature)	Adhikari B, Mishra SR, Schwarz R. Transforming Nepal’s primary health care delivery system in global health era: addressing historical and current implementation challenges. Globalization and Health. 2022 Dec;18(1):1-2.
2	Limor Adler	2022	Israel	Original article	Cross-sectional study	Adler L, Vinker S, Heymann AD, Van Poel E, Willems S, Zacay G. The effect of the COVID-19 pandemic on primary care physicians in Israel, with comparison to an international cohort: a cross-sectional study. Israel Journal of Health Policy Research. 2022 Dec;11(1):1-0.
3	Prince A. Adu	2022	Canada	Review	Scoping review	Adu PA, Stallwood L, Adebola SO, Abah T, Okpani AI. The direct and indirect impact of COVID-19 pandemic on maternal and child health services in Africa: a scoping review. Global Health Research and Policy. 2022 Dec;7(1):1-4.
4	Thamra Al Ghafri	2021	Oman	Original article	Descriptive cross-sectional study	Al Ghafri T, Al Ajmi F, Al Balushi L, Kurup PM, Al Ghamari A, Al Balushi Z, Al Fahdi F, Al Lawati H, Al Hashmi S, Al Manji A, Al Sharji A. Responses to the pandemic covid-19 in primary health care in oman: muscat experience. Oman Medical Journal. 2021 Jan;36(1):e216.
5	Ahmed Alboksmaty	2021	UK	Original article	Qualitative : semi-structured interviews	Alboksmaty A, Kumar S, Parekh R, Aylin P. Management and patient safety of complex elderly patients in primary care during the COVID-19 pandemic in the UK—Qualitative assessment. Plos one. 2021 Mar 29;16(3):e0248387.
6	Yohualli Balderas-Medina Anaya	2022	US	Policy brief	HEALTH POLICY	Anaya YB, Mota AB, Hernandez GD, Osorio A, Hayes-Bautista DE. Post-pandemic telehealth policy for primary care: an equity perspective. The Journal of the American Board of Family Medicine. 2022 May 1;35(3):588-92.
7	Enric Aragonès	2022	Spain	Original article	Cross-sectional study	Aragonès E, del Cura-González I, Hernández-Rivas L, Polentinos-Castro E, Fernández-San-Martín MI, López-Rodríguez JA, Molina-Aragonés JM, Amigo F, Alayo I, Mortier P, Ferrer M. Psychological impact of the COVID-19 pandemic on primary care workers: a cross-sectional study. British Journal of General Practice. 2022 Jul 1;72(720):e501-10.
8	Christine Ashley	2021	Australia	Original article	Qualitative descriptive study	Ashley C, James S, Williams A, Calma K, Mcinnes S, Mursa R, Stephen C, Halcomb E. The psychological well‐being of primary healthcare nurses during COVID‐19: A qualitative study. Journal of Advanced Nursing. 2021 Sep;77(9):3820-8.
9	Bishnu Bahadur Bajgain	2020	Canada	Original article	Cross-sectional study	Bajgain BB, Jackson J, Aghajafari F, Bolo C, Santana MJ. Immigrant Healthcare Experiences and Impacts During COVID-19: A Cross-Sectional Study in Alberta, Canada. Journal of Patient Experience. 2022 Jul;9:23743735221112707.
10	Mobolane Balogun	2022	Nigeria	Original article	Qualitative study	Balogun M, Banke-Thomas A, Gwacham-Anisiobi U, Yesufu V, Ubani O, Afolabi BB. Actions and adaptations implemented for maternal, newborn and child health service provision during the early phase of the COVID-19 pandemic in Lagos, Nigeria: qualitative study of health facility leaders. Annals of Global Health. 2022;88(1).
11	Prativa Baral	2021	US	Review	Rapid scoping review	Baral P. Health systems and services during COVID-19: lessons and evidence from previous crises: a rapid scoping review to inform the United Nations research roadmap for the COVID-19 recovery. International Journal of Health Services. 2021 Oct;51(4):474-93.
12	Basu P	2021	France	Review	Scoping review	Basu P, Alhomoud S, Taghavi K, Carvalho AL, Lucas E, Baussano I. Cancer screening in the coronavirus pandemic era: adjusting to a new situation. JCO global oncology. 2021 Mar;7(1):416-24.
13	Sarah L. Beebe	2022	US	Original article	Qualitative case study	Beebe SL, McKague DK, Wallington SF. COVID-19 on New Primary Care Nurse Practitioners: A Qualitative Exploration. The Journal for Nurse Practitioners. 2022 Mar 23.
14	ZACKARY BERGER	2021	US	Perspective	Perspective	Berger Z, De Jesus VA, Assoumou SA, Greenhalgh T. Long COVID and health inequities: the role of primary care. The Milbank Quarterly. 2021 Jun;99(2):519.
15	Pedro Mas Bermejo	2021	Cuba	Report	Special report	Mas Bermejo P, Sánchez Valdés L, Somarriba López L, Valdivia Onega NC, Vidal Ledo MJ, Alfonso Sánchez I, et al. Equity and the Cuban National Health System's response to COVID-19. Rev Panam Salud Publica. 2021;45:e80. 10.26633/RPSP.2021.80
16	Innocent K. Besigye	2020	Uganda	Report	Short Report	Besigye IK, Namatovu J, Mulowooza M. Coronavirus disease-2019 epidemic response in Uganda: the need to strengthen and engage primary healthcare. African Journal of Primary Health Care and Family Medicine. 2020 Jan 1;12(1):1-3.
17	Agnes Binagwaho	2022	Rwanda	Commentary	Commentary	Binagwaho A, Hirwe D, Mathewos K. Health system resilience: withstanding shocks and maintaining progress. Global Health: Science and Practice. 2022 Sep 15;10(Supplement 1).
18	Mylaine Breton	2022	Canada	Original article	Qualitative multiple case study: semi-structured interviews	Breton M, Deville-Stoetzel N, Gaboury I, Smithman MA, Kaczorowski J, Lussier MT, Haggerty J, Motulsky A, Nugus P, Layani G, Paré G. Telehealth in primary healthcare: a portrait of its rapid implementation during the COVID-19 pandemic. Healthcare Policy. 2021 Aug;17(1):73.
19	Bailey E. Bruns	2022	US	Original article	Cohort study	Bruns BE, Lorenzo-Castro SA, Hale GM. Controlling blood pressure during a pandemic: The impact of telepharmacy for primary care patients. Journal of Pharmacy Practice. 2022 Oct 27:08971900221136629.
20	Forouzan Akrami	2021	Iran	Original article	Situation analysis study	akrami F, Riazi-Isfahani S, Mahdavi hezaveh A, Ghanbari Motlagh A, najmi M, afkar M, et al . Iran’s Status of NCDs Prevention and Management Services during COVID-19 Pandemic at PHC Level. SJKU 2021; 26 (5) :50-68
21	Jafar Sadegh Tabrizi	2022	Iran	Commentary	_ -	Jafar Sadegh Tabrizi, Alireza Raeisi, Saeed Namaki, Primary Health Care and COVID-19 Pandemic in the Islamic Republic of Iran, Depiction of Health, 2022; 13(1): 1-10. magiran.com/p2451009
22	Hossein Masoumbeigi	2020	Iran	Review	Review	Masoumbeigi H, Ghanizadeh G. Challenges of Iranian Environmental Health during the COVID-19 Epidemic: Lessons for the Future. J Mil Med 2020; 22 (11) :1086-1098. ml
23	Mehrdad Kazemzadeh Atoofi	2020	Iran	Review	Mixed systematic and complementary narrative review	Kazemzadeh Atoofi M, Rezaei N, Kompani F, Shirzad F, Djalalinia S. Requirements of Mental Health Services During the COVID-19 Outbreak: A Systematic Review. IJPCP 2020; 26 (3) :264-279
24	Vida Zaroushani	2020	Iran	Review	Review	Zaroushani V. Occupational Safety and Health and Response to COVID-19 using the Fourth Industrial Revolution Technologies. J Health Saf Work 2020; 10 (4) :329-348
25	Amir Hosein Mosadeghrad	2022	Iran	Letter to Editor	-	Mosadeghrad A H. Promote COVID-19 vaccination uptake: a letter to editor. Tehran Univ Med J 2022; 80 (2) :159-160
26	Ali Mohammad Mosadeghrad	2022	Iran	Review	Scoping Review	Mosadeghrad A M, Taherkhani T, Shojaei S, Jafari M, Mohammadi S, Emamzadeh A et al . Strengthening Primary Health Care System Resilience in Covid-19 Pandemic: A Scoping Review. sjsph 2022; 20 (1) :13-24
27	Marjan Ghazi Saeedi	2021	Iran	Review	Review	Ghazi Saeedi M, Tanhapour M. Telemedicine System: A Mandatory Requirement in Today’s World. payavard 2022; 15 (5) :490-507
28	Nazi Nejat	2022	Iran	Letter to Editor	_ -	Nejat N, Borzabadi Farahani Z. COVID-19 Pandemic: Opportunities for Continuing Nursing Professional Development. J Med Educ Dev 2022; 14 (44) :1-2
29	Zahra Fotokian	2021	Iran	Letter to Editor	_-	Fotokian, Z., Mohammadkhah, F. Primary Health Care as a Strategy to Fight COVID-19 Pandemic: Letter to the Editor. Journal of Isfahan Medical School, 2021; 39(630): 470-474. https://doi.org/10.22122/jims.v39i630.14016
30	Lu M	2022	US	Original article	Cross-sectional	Lu M, Liao X. Access to care through telehealth among US Medicare beneficiaries in the wake of the COVID-pandemic.Front Public Health. 2022;10:946944
31	Luciani	2022	US	Review	Scoping review	Luciani S, Agurto I, Caixeta R, Hennis A. Prioritizing noncommunicable diseases in the Americas region in the era of COVID-19. Revista Panamericana de Salud Pública. 2022;46.
32	Ludin N	2022	New Zealand	Original article	Mixed method	Ludin N, Holt-Quick C, Hopkins S, Stasiak K, Hetrick S, Warren J, Cargo T. A Chatbot to Support Young People During the COVID-19 Pandemic in New Zealand: Evaluation of the Real-World Rollout of an Open Trial. Journal of Medical Internet Research. 2022 Nov 4;24(11):e38743.
33	Saxenian	2022	US	Review	Review	Saxenian H, Alkenbrack S, Attaran MF, Barcarolo J, Brenzel L, Brooks A, Ekeman E, Griffiths UK, Rozario S, Maele NV, Ranson MK. Sustainable financing for Immunization Agenda 2030. Vaccine. 2022 Dec 2.
34	Savoy A	2022	US	Original article	Intervention	Savoy A, Patel H, Shahid U, Offner AD, Singh H, Giardina TD, Meyer AN. Electronic Co-design (ECO-design) Workshop for Increasing Clinician Participation in the Design of Health Services Interventions: Participatory Design Approach. JMIR Human Factors. 2022 Sep 22;9(3):e37313.
35	Saura Llamas	2021	Spain	Rview	Review	Llamas S, MP AP, Felipe P. Patient safety training and a safe teaching in primary care. Atencion Primaria. 2021 Dec 1;53:102199-.
36	Saso A	2020	UK	Original article	Quantitative	Saso A, Skirrow H, Kampmann B. Impact of COVID-19 on immunization services for maternal and infant vaccines: results of a survey conducted by imprint—the immunising pregnant women and infants network. Vaccines. 2020 Sep 22;8(3):556.
37	Reges O	2022	Israel	Original article	Quantitative	Reges O, Feldhamer I, Wolff Sagy Y, Lavie G. Factors Associated with Using Telemedicine in the Primary Care Clinics during the COVID-19 Pandemic in Israel. International Journal of Environmental Research and Public Health. 2022 Oct 14;19(20):13207.
38	Reath J	2022	Australia	Review	Review	Reath J, Lau P, Lo W, Trankle S, Brooks M, Shahab Y, Abbott P. Strengthening learning and research in health equity–opportunities for university departments of primary health care and general practice. Australian Journal of Primary Health. 2022 Nov 8.
39	Saab MM	2022	Ireland	Original article	Qualitative	Saab MM, O’Driscoll M, FitzGerald S, Sahm LJ, Leahy-Warren P, Noonan B, Kilty C, Lyons N, Burns HE, Kennedy U, Lyng Á. Primary healthcare professionals’ perspectives on patient help-seeking for lung cancer warning signs and symptoms: a qualitative study. BMC Primary Care. 2022 Dec;23(1):1-5.
40	Sandhu HS	2022	Canada	Original article	Qualitative: interview	Sandhu HS, Smith RW, Jarvis T, O'Neill M, Di Ruggiero E, Schwartz R, Rosella LC, Allin S, Pinto AD. Early Impacts of the COVID-19 Pandemic on Public Health Systems and Practice in 3 Canadian Provinces From the Perspective of Public Health Leaders: A Qualitative Study. Journal of Public Health Management and Practice. 2022 Nov 8;28(6):702-11.
41	Ma L,	2022	China	Original article	Quantitative	Ma L, Han X, Ma Y, Yang Y, Xu Y, Liu D, Yang W, Feng L. Decreased influenza vaccination coverage among Chinese healthcare workers during the COVID-19 pandemic. Infectious Diseases of Poverty. 2022 Oct 10;11(05):63-73.
42	Murphy M	2021	UK	Original article	Mixed-methods study	Murphy M, Scott LJ, Salisbury C, Turner A, Scott A, Denholm R, Lewis R, Iyer G, Macleod J, Horwood J. Implementation of remote consulting in UK primary care following the COVID-19 pandemic: a mixed-methods longitudinal study. British Journal of General Practice. 2021 Mar 1;71(704):e166-77.
43	Piché-Renaud PP	2021	Canada	Original article	Quantitative	Piché-Renaud PP, Ji C, Farrar DS, Friedman JN, Science M, Kitai I, Burey S, Feldman M, Morris SK. Impact of the COVID-19 pandemic on the provision of routine childhood immunizations in Ontario, Canada. Vaccine. 2021 Jul 13;39(31):4373-82.
44	Maria AR	2022	Portugal	Original article	Qualitative: interview	Maria AR, Serra H, Heleno B. Teleconsultations and their implications for health care: A qualitative study on patients’ and physicians’ perceptions. International Journal of Medical Informatics. 2022 Jun 1;162:104751.
45	Mills WR	2020	US	Original article	Quantitative	Mills WR, Buccola JM, Sender S, Lichtefeld J, Romano N, Reynolds K, Price M, Phipps J, White L, Howard S. Home-based primary care led-outbreak mitigation in assisted living facilities in the first 100 days of coronavirus disease 2019. Journal of the American Medical Directors Association. 2020 Jul 1;21(7):951-3.
46	Mirsky JB	2021	US	Review	Review	Mirsky JB, Thorndike AN. Virtual group visits: hope for improving chronic disease management in primary care during and after the COVID-19 pandemic. American Journal of Health Promotion. 2021 Sep;35(7):904-7.
47	Neves AL	2021	UK	Review	Review	Neves AL, Li E, Gupta PP, Fontana G, Darzi A. Virtual primary care in high-income countries during the COVID-19 pandemic: Policy responses and lessons for the future. European Journal of General Practice. 2021 Jan 1;27(1):241-7.
48	Otu A	2021	Nigeria	Original article	Quantitative	Otu A, Okuzu O, Ebenso B, Effa E, Nihalani N, Olayinka A, Yaya S. Introduction of mobile health tools to support COVID-19 training and surveillance in Ogun State Nigeria. Frontiers in Sustainable Cities. 2021 Mar 5;3:638278.
49	Anita Mfuh	2021	Nigeria	Original article	Quantitative	Lukong AM, Jafaru Y. Covid-19 pandemic challenges, coping strategies and resilience among healthcare workers: A multiple linear regression analysis. African Journal of Health, Nursing and Midwifery. 2021;4:16-27.
50	Anja Rieckert	2020	Netherlands	Review	Review	Rieckert A, Schuit E, Bleijenberg N, et al. How can we build and maintain the resilience of our health care professionals during COVID-19? Recommendations based on a scoping review. BMJ Open 2021;11:e043718. doi:10.1136/ bmjopen-2020-043718
51	Sagan A	2020	UK	Review	Review	Sagan A, Thomas S, McKee M, Karanikolos M, Azzopardi-Muscat N, de la Mata I, Figueras J, World Health Organization. COVID-19 and health systems resilience: lessons going forwards. Eurohealth. 2020;26(2):20-4.
52	Etienne CF	2020	US	commentary	_	Etienne CF, Fitzgerald J, Almeida G, Birmingham ME, Brana M, Bascolo E, Cid C, Pescetto C. COVID-19: transformative actions for more equitable, resilient, sustainable societies and health systems in the Americas. BMJ Global Health. 2020 Aug 1;5(8):e003509.
53	Eze-Emiri C	2022	Nigeria	Original article	Retrospective cohort	Eze-Emiri C, Patrick F, Igwe E, Owhonda G. Retrospective study of COVID-19 outcomes among healthcare workers in Rivers State, Nigeria. BMJ open. 2022 Nov 1;12(11):e061826.
54	Fadul N	2021	US	Report	Short Communication	Fadul N, Regan N, Kaddoura L, Swindells S. A midwestern academic HIV clinic operation during the COVID-19 pandemic: implementation strategy and preliminary outcomes. Journal of the International Association of Providers of AIDS Care (JIAPAC). 2021 Sep 2;20:23259582211041423.
55	Fang Y	2022	Singapore	Original article	cross sectional survey (qualitative)	Fang Y, Soljak M, Tan SL, Peckham S, Tan TL, Smith HE. General practitioners’ views on retaining Singapore’s primary care doctors: a cross-sectional survey and qualitative analysis. BMC primary care. 2022 Dec;23(1):1-1.
56	Abd El Kader AI	2022	Egypt	Original article	Explanatory research design	Abd El Kader AI, Faramawy MA. COVID-19 anxiety and organizational commitment among front line nurses: Perceived role of nurse managers' caring behavior. Nursing Practice Today. 2022;9(1):X-.
57	Farsalinos K	2021	Greece	Original article	Qualitative, Case study	Farsalinos K, Poulas K, Kouretas D, Vantarakis A, Leotsinidis M, Kouvelas D, Docea AO, Kostoff R, Gerotziafas GT, Antoniou MN, Polosa R. Improved strategies to counter the COVID-19 pandemic: Lockdowns vs. primary and community healthcare. Toxicology Reports. 2021 Jan 1;8:1-9.
58	Fatima R	2021	Pakistan	Original article	Qualitative, case study	Fatima R, Akhtar N, Yaqoob A, Harries AD, Khan MS. Building better tuberculosis control systems in a post-COVID world: learning from Pakistan during the COVID-19 pandemic. International Journal of Infectious Diseases. 2021 Dec 1;113:S88-90.
59	Ferenčina J	2022	Slovenia	Original article	Observational	Ferenčina J, Tomšič V. COVID-19 clinic as a basis of quality primary health care in the light of the pandemic–an observational study. Med Glas (Zenica). 2022 Feb 1;19(1).
60	Ferorelli D	2020	Italy	commentary	-	Ferorelli D, Nardelli L, Spagnolo L, Corradi S, Silvestre M, Misceo F, Marrone M, Zotti F, Mandarelli G, Solarino B, Dell’Erba A. Medical legal aspects of telemedicine in Italy: application fields, professional liability and focus on care services during the COVID-19 health emergency. Journal of Primary Care & Community Health. 2020 Dec;11:2150132720985055.
61	Ferreira NN,	2022	Brazil	Original article	Prospective observational cohort study	Ferreira NN, Garibaldi PM, Moraes GR, Moura JC, Klein TM, Machado LE, Scofoni LF, Haddad SK, Calado RT, Covas DT, Fonseca BA. The impact of an enhanced health surveillance system for COVID-19 management in Serrana, Brazil. Public Health in Practice. 2022 Dec 1;4:100301.
62	Fitzpatrick K	2022	Canada	Review	Rapid review	Fitzpatrick K, Sehgal A, Montesanti S, Pianarosa E, Barnabe C, Heyd A, Kleissen T, Crowshoe L. Examining the role of Indigenous primary healthcare across the globe in supporting populations during public health crises. Global Public Health. 2022 Mar 24:1-29.
63	Florea M	2021	Romania	Original article	Cross sectional	Florea M, Lazea C, Gaga R, Sur G, Lotrean L, Puia A, Stanescu AM, Lupsor-Platon M, Florea H, Sur ML. Lights and shadows of the perception of the use of telemedicine by Romanian family doctors during the COVID-19 pandemic. International journal of general medicine. 2021;14:1575.
64	Franck E	2022	Belgium	Original article	Survey	Franck E, Goossens E, Haegdorens F, Geuens N, Portzky M, Tytens T, Dilles T, Beeckman K, Timmermans O, Slootmans S, Van Rompaey B. Role of resilience in healthcare workers’ distress and somatization during the COVID‐19 pandemic: A cross‐sectional study across Flanders, Belgium. Nursing open. 2022 Mar;9(2):1181-9.
65	Franzosa E	2021	US	Original article	Qualitative, semi-structured interviews	Franzosa E, Gorbenko K, Brody AA, Leff B, Ritchie CS, Kinosian B, Ornstein KA, Federman AD. “At home, with care”: lessons from New York City home‐based primary care practices managing COVID‐19. Journal of the American Geriatrics Society. 2021 Feb;69(2):300-6.
66	Freed SL	2022	US	Notes From the Field	-	Freed SL, Thiele D, Gardner M, Myers E. COVID-19 Evaluation and Testing Strategies in a Federally Qualified Health Center. American Journal of Public Health. 2022 Jun;112(S3):S284-7.
67	Frost R	2020	UK	Letter to Editor	_	Frost R, Nimmons D, Davies N. Using remote interventions in promoting the health of frail older persons following the COVID-19 lockdown: challenges and solutions. Journal of the American Medical Directors Association. 2020 Jul;21(7):992.
68	Fulmer T	2021	US	Commentary	_	Fulmer T, Reuben DB, Auerbach J, Fick DM, Galambos C, Johnson KS. Actualizing Better Health And Health Care For Older Adults: Commentary describes six vital directions to improve the care and quality of life for all older Americans. Health Affairs. 2021 Feb 1;40(2):219-25.
69	Gallardo-Rincón H	2022	Mexico	Original article	Cross sectional-Quantitative	Gallardo-Rincón H, Gascon JL, Martínez-Juárez LA, Montoya A, Saucedo-Martínez R, Rosales RM, Tapia-Conyer R. MIDO COVID: A digital public health strategy designed to tackle chronic disease and the COVID-19 pandemic in Mexico. Plos one. 2022 Nov 17;17(11):e0277014.
70	Giannopoulou I,	2020	Greece	Perspective	-_	Giannopoulou I, Tsobanoglou GO. COVID-19 pandemic: challenges and opportunities for the Greek health care system. Irish journal of psychological medicine. 2020 Sep;37(3):226-30.
71	Golden EA	2021	US	Original article	Quantitative , Cross sectional	Golden EA, Zweig M, Danieletto M, Landell K, Nadkarni G, Bottinger E, Katz L, Somarriba R, Sharma V, Katz CL, Marin DB. A resilience-building app to support the mental health of health care workers in the COVID-19 era: Design process, distribution, and evaluation. JMIR Formative Research. 2021 May 5;5(5):e26590.
72	Golechha M	2022	India	Original article	Qualitative: In depth interview	Golechha M, Bohra T, Patel M, Khetrapal S. Healthcare worker resilience during the COVID‐19 pandemic: A qualitative study of primary care providers in India. World medical & health policy. 2022 Mar;14(1):6-18.
73	Gomez T	2021	US	Original article	Qualitative: In depth interview	Gomez T, Anaya YB, Shih KJ, Tarn DM. A qualitative study of primary care physicians’ experiences with telemedicine during COVID-19. The Journal of the American Board of Family Medicine. 2021 Feb 1;34(Supplement):S61-70.
74	Gómez-Restrepo C	2022	US	Original article	Survey	Gómez-Restrepo C, Cepeda M, Torrey WC, Suarez-Obando F, Uribe-Restrepo JM, Park S, Acosta MP, Camblor PM, Castro SM, Aguilera-Cruz J, González L. Perceived access to general and mental healthcare in primary care in Colombia during COVID-19: A cross-sectional study. Frontiers in public health. 2022:3124.
75	Gong F	2021	China	Perspective	-_	Gong F, Hu G, Lin H, Sun X, Wang W. Integrated healthcare systems response strategies based on the Luohu model during the COVID-19 epidemic in Shenzhen, China. International Journal of Integrated Care. 2021 Jan;21(1).
76	Goodyear-Smith F	2022	New Zealand	Report	-	Goodyear-Smith F, Kidd M, Oseni TI, Nashat N, Mash R, Akman M, Phillips RL, van Weel C. International examples of primary care COVID-19 preparedness and response: a comparison of four countries. Family Medicine and Community Health. 2022;10(2).
77	Gray C	2022	US	Original article	Interview	Gray C, Ambady L, Chao S, Smith W, Yoon J. Virtual Management of Chronic Conditions During the COVID-19 Pandemic: Insights From Primary Care Providers and Clinical Pharmacists. Military Medicine. 2022 Sep 28.
78	Haase CB	2021	Denmark	commentary	-	Haase CB, Bearman M, Brodersen J, Hoeyer K, Risor T. ‘You should see a doctor’, said the robot: Reflections on a digital diagnostic device in a pandemic age. Scandinavian Journal of Public Health. 2021 Feb;49(1):33-6.
79	Haldane V	2021	Switzerland	Review	Review	Haldane V, De Foo C, Abdalla SM, Jung AS, Tan M, Wu S, Chua A, Verma M, Shrestha P, Singh S, Perez T. Health systems resilience in managing the COVID-19 pandemic: lessons from 28 countries. Nature Medicine. 2021 Jun;27(6):964-80.
80	Haldane V	2022	Philippine	Original article	Qualitative: in-depth interviews	Haldane V, Dodd W, Kipp A, Ferrolino H, Wilson K, Servano D, Lau LL, Wei X. Extending health systems resilience into communities: a qualitative study with community-based actors providing health services during the COVID-19 pandemic in the Philippines. BMC health services research. 2022 Dec;22(1):1-2.
81	Haldane V	2020	Canada	Review	Rapid review	Haldane V, Zhang Z, Abbas RF, Dodd W, Lau LL, Kidd MR, Rouleau K, Zou G, Chao Z, Upshur RE, Walley J. National primary care responses to COVID-19: a rapid review of the literature. BMJ open. 2020 Dec 1;10(12):e041622.
82	Hariyani N,	2022	Indonesia	Review	Narrative review	Hariyani N, Shanbhag N, Wijayati EW, Prananta AW, Setyowati D, Palupi R. Teledentistry and online referral system in Indonesian primary health care center during the COVID-19 pandemic: A narrative review. Journal of International Society of Preventive and Community Dentistry. 2022 Jul 1;12(4):385.
83	Harzheim E	2020	Brazil	Prespective	_	Harzheim E, Martins C, Wollmann L, Pedebos LA, Faller LD, Marques MD, Minei TS, Cunha CR, Telles LF, Moura LJ, Leal MH. Federal actions to support and strengthen local efforts to combat COVID-19: Primary Health Care (PHC) in the driver's seat. Ciência & Saúde Coletiva. 2020 Jun 5;25:2493-7.
84	Haun JN	2021	US	Original article	Mixed-method descriptive study	Haun JN, Cotner BA, Melillo C, Panaite V, Messina W, Patel-Teague S, Zilka B. Proactive integrated virtual healthcare resource use in primary care. BMC health services research. 2021 Dec;21(1):1-4.
85	Hearnshaw S	2021	UK	Evaluating health services	Donabedian’s conceptual model for evaluating health services	Hearnshaw S, Serban S, Mohammed I, Zubair A, Jaswal D, Grant S. A Local Dental Network Approach to the COVID-19 Pandemic: Innovation Through Collaboration. Primary Dental Journal. 2021 Mar;10(1):33-9.
86	Hernández Rincón EH	2021	Colombia	Review	Review	Hernández Rincón EH, Pimentel González JP, Aramendiz Narváez MF, Araujo Tabares RA, Roa González JM. Description and analysis of primary care-based COVID-19 interventions in Colombia. Medwave. 2021 Jul 4:e8147-.
87	Hincapié MA	2020	Colombia	Review	Scoping review	Hincapié MA, Gallego JC, Gempeler A, Piñeros JA, Nasner D, Escobar MF. Implementation and usefulness of telemedicine during the COVID-19 pandemic: a scoping review. Journal of primary care & community health. 2020 Dec;11:2150132720980612.
88	Hoeft TJ,	2021	US	Commentary	_-	Hoeft TJ, Hessler D, Francis D, Gottlieb LM. Applying lessons from behavioral health integration to social care integration in primary care. The Annals of Family Medicine. 2021 Jul 1;19(4):356-61.
89	Hundal GS	2021	US	Original article	Interview	Hundal GS, Thiyagarajan S, Alduraibi M, Laux CM, Furterer SL, Cudney EA, Antony J. Lean Six Sigma as an organizational resilience mechanism in health care during the era of COVID-19. International Journal of Lean Six Sigma. 2021 Jan 25.
90	Hussein ES	2022	Saudi Arabia	Original article	Cross sectional	Hussein ES, Al-Shenqiti AM, Ramadan RM. Applications of Medical Digital Technologies for Noncommunicable Diseases for Follow-Up during the COVID-19 Pandemic. International Journal of Environmental Research and Public Health. 2022 Oct 4;19(19):12682.
91	Ibragimov K	2022	France	Original article	Mixed method	Ibragimov K, Palma M, Keane G, Ousley J, Crowe M, Carreño C, Casas G, Mills C, Llosa A. Shifting to Tele-Mental Health in humanitarian and crisis settings: an evaluation of Médecins Sans Frontières experience during the COVID-19 pandemic. Conflict and health. 2022 Dec;16(1):1-5.
92	Ismail SA	2022	UK	Review	Review	Ismail SA, Lam ST, Bell S, Fouad FM, Blanchet K, Borghi J. Strengthening vaccination delivery system resilience in the context of protracted humanitarian crisis: a realist-informed systematic review. BMC Health Services Research. 2022 Dec;22(1):1-21.
93	Johansen AS	2021	Armenia	Development	-	Johansen AS, Shriwise A, Lopez-Acuna D, Vracko P. Strengthening the primary health care response to COVID-19: an operational tool for policymakers. Primary health care research & development. 2021;22.
94	Jonnagaddala J	2021	Australia	Review	Rapid scoping review	Jonnagaddala J, Godinho MA, Liaw ST. From telehealth to virtual primary care in Australia? a rapid scoping review. International Journal of Medical Informatics. 2021 Jul 1;151:104470.
95	Karim SI	2020	Saudi Arabia	commentary	-	Karim SI, Irfan F, Batais MA. Becoming virtual: a preliminary experience of outpatient primary care during COVID-19 pandemic. The Pan African Medical Journal. 2020;37.
96	Kelly F	2021	South Africa	Original article	Quantitative	Kelly F, Uys M, Bezuidenhout D, Mullane SL, Bristol C. Improving Healthcare Worker Resilience and Well-Being During COVID-19 Using a Self-Directed E-Learning Intervention. Frontiers in Psychology. 2021;12.
97	Kim AY	2021	Korea	Original article	Case study	Kim AY, Choi WS. Considerations on the implementation of the telemedicine system encountered with stakeholders' resistance in COVID-19 pandemic. Telemedicine and e-Health. 2021 May 1;27(5):475-80.
98	Kinder K	2021	Germany	Report	-	Kinder K, Bazemore A, Taylor M, Mannie C, Strydom S, George J, Goodyear-Smith F. Integrating primary care and public health to enhance response to a pandemic. Primary Health Care Research & Development. 2021;22.
99	Knop M	2021	Germany	Original article	Qualitative: Interview	Knop M, Mueller M, Niehaves B. Investigating the use of telemedicine for digitally mediated delegation in team-based primary care: Mixed methods study. Journal of medical Internet research. 2021 Aug 26;23(8):e28151.
100	Kumpunen S	2022	UK	Report	-	Kumpunen S, Webb E, Permanand G, Zheleznyakov E, Edwards N, van Ginneken E, Jakab M. Transformations in the landscape of primary health care during COVID-19: themes from the European region. Health Policy. 2022 May 1;126(5):391-7.
101	Lahariya C	2020	India	Original article	Case study	Lahariya C. Health & wellness centers to strengthen primary health care in India: Concept, progress and ways forward. The Indian Journal of Pediatrics. 2020 Nov;87(11):916-29.
102	Lamberti-Castronuovo A	2022	Italy	Review	Review	Lamberti-Castronuovo A, Valente M, Barone-Adesi F, Hubloue I, Ragazzoni L. Primary health care disaster preparedness: A review of the literature and the proposal of a new framework. International Journal of Disaster Risk Reduction. 2022 Sep 2:103278.
103	Lauriola P	2022	UK	Analysis	-	Lauriola P, Martín-Olmedo P, Leonardi GS, Bouland C, Verheij R, Dückers ML, Van Tongeren M, Laghi F, Van Den Hazel P, Gokdemir O, Segredo E. On the importance of primary and community healthcare in relation to global health and environmental threats: lessons from the COVID-19 crisis. BMJ global health. 2021 Mar 1;6(3):e004111.
104	Levy P	2021	US	Original article	Case study	Levy P, McGlynn E, Hill AB, Zhang L, Korzeniewski SJ, Foster B, Criswell J, O’Brien C, Dawood K, Baird L, Shanley CJ. From pandemic response to portable population health: A formative evaluation of the Detroit mobile health unit program. Plos one. 2021 Nov 30;16(11):e0256908.
105	Li D	2021	UK	Original article	Case study	Li D, Howe AC, Astier-Peña MP. Primary health care response in the management of pandemics: Learnings from the COVID-19 pandemic. Atencion Primaria. 2021 Dec 1;53:102226.
106	Liaw ST	2021	Australia	Original article	Case study	Liaw ST, Kuziemsky C, Schreiber R, Jonnagaddala J, Liyanage H, Chittalia A, Bahniwal R, He JW, Ryan BL, Lizotte DJ, Kueper JK. Primary care informatics response to Covid-19 pandemic: adaptation, progress, and lessons from four countries with high ICT development. Yearbook of medical informatics. 2021 Aug;30(01):044-55.
107	Liddy C	2022	Canada	Original article	Cross sectional	Liddy C, Singh J, Mitchell R, Guglani S, Keely E. How one eConsult service is addressing emerging COVID-19 questions. The Journal of the American Board of Family Medicine. 2022 May 1;35(3):601-4.
108	Litke N	2022	Germany	Original article	Qualitative	Litke N, Weis A, Koetsenruijter J, Fehrer V, Koeppen M, Kuemmel S, Szecsenyi J, Wensing M. Building resilience in German primary care practices: a qualitative study. BMC primary care. 2022 Dec;23(1):1-4.
109	Ivone Evangelista Cabral	2021	Brazil	Review	Document Review	Cabral IE, Pestana-Santos M, Ciuffo LL, Nunes YDR, Lomba MLLF. Child health vulnerabilities during the COVID-19 pandemic in Brazil and Portugal. Rev Lat Am Enfermagem. 2021;29:e3422. Published 2021 Jul 2. doi:10.1590/1518-8345.4805.3422
110	José Calvo-Paniagua	2022	Spain	Original article	Action Research	Calvo-Paniagua J, Díaz-Arribas MJ, Valera-Calero JA, et al. A tele-health primary care rehabilitation program improves self-perceived exertion in COVID-19 survivors experiencing Post-COVID fatigue and dyspnea: A quasi-experimental study. PLoS One. 2022;17(8):e0271802. Published 2022 Aug 4. doi:10.1371/journal.pone.0271802
111	Luz Elena Ojeda Carmona	2020	Brazil	Review	Document Reveiw	Carmona LE, Nielfa MD, Alvarado AL. The Covid-19 pandemic seen from the frontline. International braz j urol. 2020 Jul 27;46:181-94.
112	Ianka Cristina Celuppi	2022	Brazil	Original article	Qualitative	Celuppi IC, Meirelles BH, Lanzoni GM, Geremia DS, Metelski FK. Management in the care of people with HIV in primary health care in times of the new coronavirus. Revista de Saúde Pública. 2022 Apr 1;56:13.
113	Lea Chaiban	2022	Lebanon	Original article	Qualitative	Chaiban L, Benyaich A, Yaacoub S, Rawi H, Truppa C, Bardus M. BMC Health Services Research. 2022;22(1).
114	Alyssa Yenyi Chan	2022	Singapore	Original article	Qualitative	Chan AY, Ting C, Chan LG, Hildon ZJL. Human Resources for Health. 2022;20(1).
115	Kevin Chen	2022	US	Original article	Cross‐sectional	Chen K, Davoodi NM, Strauss DH, Li M, Jiménez FN, Guthrie KM, et al. Journal of Applied Gerontology. 2022;41(11):2282-95.
116	Cristelle Chow	2021	Singapore	Original article	Qualitative	Chow C, Goh SK, Tan CS, Wu HK, Shahdadpuri R. Enhancing frontline workforce volunteerism through exploration of motivations and impact during the COVID-19 pandemic. International Journal of Disaster Risk Reduction. 2021 Dec 1;66:102605.
117	Alvin Qijia Chua	2020	Singapore	commentary	Commentary	Chua AQ, Tan MMJ, Verma M, Han EKL, Hsu LY, Cook AR, et al. BMJ global health. 2020;5(9):e003317.
118	Claudia Corwin	2021	US	commentary	Commentary	Corwin C, Sinnwell E, Culp K. Journal of agromedicine. 2021;26(3):346-51.
119	Ivana T. Croghan	2021	US	Original article	Survey	Croghan IT, Chesak SS, Adusumalli J, Fischer KM, Beck EW, Patel SR, Ghosh K, Schroeder DR, Bhagra A. Stress, resilience, and coping of healthcare workers during the COVID-19 pandemic. Journal of Primary Care & Community Health. 2021 Apr;12:21501327211008448.
120	Katrien Danhieux,	2020	Belgium	Original article	Qualitative	Danhieux K, Buffel V, Pairon A, Benkheil A, Remmen R, Wouters E, et al. BMC FAMILY PRACTICE. 2020;21(1).
121	Melissa Daou	2021	Lebanon	Conference article	Qualitative	Daou M, Helou S, El Helou J, El Hachem C, El Helou E. Stud Health Technol Inform. 2022;290:937-41.
122	Jan De Maeseneer	2021	UK	Commentary	Commentary	De Maeseneer J. Prim Health Care Res Dev. 2021;22:e73.
123	Peter A. Delobelle	2022	South Africa	Original article	Mixed methods	Delobelle PA, Abbas M, Datay I, De Sa A, Levitt N, Schouw D, et al. Afr J Prim Health Care Fam Med. 2022;14(1):e1-e7.
124	Lea den Broeder	2022	Netherlands	Review	Review	Den Broeder L, South J, Rothoff A, Bagnall AM, Azarhoosh F, van der Linden G, et al. HEALTH PROMOTION INTERNATIONAL. 2022;37(2).
125	Jean-Louis Denis	2020	Canada	Commentary	Commentary	Denis JL, Potvin L, Rochon J, Fournier P, Gauvin L. Can J Public Health. 2020;111(6):912-20.
126	Jane Desborough	2021	Australia	Review	Review	Desborough J, Dykgraaf SH, Phillips C, Wright M, Maddox R, Davis S, et al. Fam Pract. 2021;38(6):811-25.
127	N.R. DeTore	2022	US	Original article	Survey	DeTore NR, Sylvia L, Park ER, Burke A, Levison JH, Shannon A, et al. Journal of psychiatric research. 2022;146:228-33.
128	Jennifer E. DeVoe	2020	US	Commentary	-	.DeVoe JE, Cheng A, Krist A. JAMA Health Forum. 2020;1(4):e200423.
129	Riyanti Djalante	2020	Japan	Review	Review	Djalante R, Shaw R, DeWit A. Building resilience against biological hazards and pandemics: COVID-19 and its implications for the Sendai Framework. Progress in disaster science. 2020 Apr 1;6:100080..
130	Aleksandar Džakula	2022	Croatia	Review	Review	Džakula A, Banadinović M, Lovrenčić IL, Vajagić M, Dimova A, Rohova M, et al. Health Policy. 2022;126(5):456-64.
131	Marion Eisele	2021	Germany	Original article	Survey	Eisele M, Pohontsch NJ, Scherer M. Strategies in primary care to face the SARS-CoV-2/COVID-19 pandemic: an online survey. Frontiers in Medicine. 2021 Jun 2;8:613537.
132	Diana C. Schow	2022	US	Original article	Qualitative	Schow DC, Thomson A, Trusty WT, Buchi-Fotre L. Use of a research as intervention approach to explore telebehavioral health services during the COVID-19 pandemic in Southeastern Idaho. Journal of Primary Care & Community Health. 2022 Jan;13:21501319211072998.
133	Mark Segal	2022	US	Original article	Qualitative	Segal M, Giuffrida P, Possanza L, Bucciferro D. The Critical Role of Health Information Technology in the Safe Integration of Behavioral Health and Primary Care to Improve Patient Care. J Behav Health Serv Res. 2022 Apr;49(2):221-230. https://doi.org/10.1007/s11414-021-09774-0. Epub 2021 Oct 19. PMID: 34668115; PMCID: PMC8525847.
134	Selick A	2022	Canada	Original article	Qualitative	Selick A, Durbin J, Hamdani Y, Rayner J, Lunsky Y. Accessibility of Virtual Primary Care for Adults With Intellectual and Developmental Disabilities During the COVID-19 Pandemic: Qualitative Study. JMIR Form Res 2022;6(8):e38916. URL: https://formative.jmir.org/2022/8/e38916. 10.2196/38916
135	Sachin Shailendra Shah	2021	UK	Original article	Prospective observational	Shah, S.S., Safa, A., Johal, K. et al. A prospective observational real world feasibility study assessing the role of app-based remote patient monitoring in reducing primary care clinician workload during the COVID pandemic. BMC Fam Pract 22, 248 (2021). https://doi.org/10.1186/s12875-021-01594-7
136	Babar Tasneem Shaikh	2021	Pakistan	Review	Systematic review	Shaikh BT. Strengthening health system building blocks: configuring post-COVID-19 scenario in Pakistan. Primary Health Care Research & Development. Cambridge University Press; 2021;22:e9.
137	Lu-shao-bo Sh	2022	China	Original article	Descriptive analysis	Shi LS, Xu RH, Xia Y, Chen DX, Wang D. The Impact of COVID-19-Related Work Stress on the Mental Health of Primary Healthcare Workers: The Mediating Effects of Social Support and Resilience. Front Psychol. 2022 Jan 21;12:800183. https://doi.org/10.3389/fpsyg.2021.800183. PMID: 35126252; PMCID: PMC8814425.
138	Jung-ha Kim	2021	Korea	Original article	Qualitative: Delphi	Shin WY, Kim C, Lee SY, Lee W, Kim JH. Role of primary care and challenges for public–private cooperation during the coronavirus disease 2019 pandemic: An expert Delphi study in South Korea. Yonsei medical journal. 2021 Jul 7;62(7):660.
139	Emil Larus Sigurdsson	2020	Iceland	Original article	Descriptive and analytical	Sigurdsson EL, Blondal AB, Jonsson JS, Tomasdottir MO, Hrafnkelsson H, Linnet K, Sigurdsson JA. How primary healthcare in Iceland swiftly changed its strategy in response to the COVID-19 pandemic. BMJ Open. 2020 Dec 7;10(12):e043151. https://doi.org/10.1136/bmjopen-2020-043151. PMID: 33293329; PMCID: PMC7722808.
140	Line Silsand	2021	Norway	Original article	Qualitative	Silsand L, Severinsen GH, Berntsen G. Preservation of Person-Centered care through videoconferencing for patient follow-up during the COVID-19 pandemic: case study of a multidisciplinary care team. JMIR formative research. 2021 Mar 5;5(3):e25220.
141	Silva C,	2022	Brazil	Review	Scoping review	Silva CRDV, Lopes RH, de Goes Bay O Jr, Martiniano CS, Fuentealba-Torres M, Arcêncio RA, Lapão LV, Dias S, Uchoa SADC. Digital Health Opportunities to Improve Primary Health Care in the Context of COVID-19: Scoping Review. JMIR Hum Factors. 2022 May 31;9(2):e35380. https://doi.org/10.2196/35380. PMID: 35319466; PMCID: PMC9159467.
142	Maria João Silva	2021	Portugal	Original article	Cross-sectional	Silva MJ, Santos P. The Impact of Health Literacy on Knowledge and Attitudes towards Preventive Strategies against COVID-19: A Cross-Sectional Study. Int J Environ Res Public Health. 2021 May 19;18(10):5421. https://doi.org/10.3390/ijerph18105421. PMID: 34069438; PMCID: PMC8159089.
143	Andrew Smaggus	2021	Canada	Original article	Qualitative: content analysis	Smaggus, A., Long, J., Ellis, L. A., Clay-Williams, R., Braithwaite, J. Government Actions and Their Relation to Resilience in Healthcare During the COVID-19 Pandemic in New South Wales, Australia and Ontario, Canada. International Journal of Health Policy and Management, 2022; 11(9): 1682-1694. https://doi.org/10.34172/ijhpm.2021.67
144	Solari-Twadell	2021	US	Original article	Qualitative	Solari-Twadell PA, Flinter M, Rambur B, Renda S, Witwer S, Vanhook P, Poghosyan L. The impact of the COVID-19 pandemic on the future of telehealth in primary care. Nurs Outlook. 2022 Mar-Apr;70(2):315-322. https://doi.org/10.1016/j.outlook.2021.09.004. Epub 2021 Sep 24. PMID: 34763897; PMCID: PMC8461221.
145	Carlos Dornels Freire de SOUZA	2020	Brazil	Policy brief	_	Souza CD, Gois-Santos VT, Correia DS, Martins-Filho PR, Santos VS. The need to strengthen Primary Health Care in Brazil in the context of the COVID-19 pandemic. Brazilian oral research. 2020 May 11;34:e047.
146	Sandra Stengel*	2022	Germany	Original article	Qualitative	Stengel, S., Roth, C., Breckner, A. et al. Resilience of the primary health care system – German primary care practitioners’ perspectives during the early COVID-19 pandemic. BMC Prim. Care 23, 203 (2022). https://doi.org/10.1186/s12875-022-01786-9
147	Kadri Suija	2022	Estonia	Original article	Qualitative: interview study	Suija, K., Mardo, L.A., Laidoja, R. et al. Experiences and expectation with the use of health data: a qualitative interview study in primary care. BMC Prim. Care 23, 159 (2022). https://doi.org/10.1186/s12875-022-01764-1
148	Erin E. Sullivan	2022	US	Original article	Mixed methods	Sullivan EE, McKinstry D, Adamson J, Hunt L, Phillips RS, Linzer M. Burnout Among Missouri Primary Care Clinicians in 2021: Roadmap for Recovery? Mo Med. 2022 Jul-Aug;119(4):397-400. PMID: 36118800; PMCID: PMC9462904.
149	Erin E. Sullivan	2020	US	Commentary	_-	Sullivan, E.E., Phillips, R.S. Sustaining primary care teams in the midst of a pandemic. Isr J Health Policy Res 9, 77 (2020). https://doi.org/10.1186/s13584-020-00434-w
150	Abida Sultana	2021	Bangladesh	Letter to Editor	-_	Sultana A, Bhattacharya S, Hossain MM. COVID-19 and primary care: a critical need for strengthening emergency preparedness across health systems. Journal of Family Medicine and Primary Care. 2021 Jan 1;10(1):584-5.
151	T. Sundararaman	2021	India	Review	Review	Sundararaman T, Muraleedharan VR, Ranjan A. Pandemic resilience and health systems preparedness: lessons from COVID-19 for the twenty-first century. J Soc Econ Dev. 2021 Jan 6;23(Suppl 2):1-11. https://doi.org/10.1007/s40847-020-00133-x. Epub ahead of print. PMID: 34720480; PMCID: PMC7786882.
152	Nanae Tanemura	2022	Japan	Original article	Online survey	Tanemura N, Chiba T. The usefulness of a checklist approach-based confirmation scheme in identifying unreliable COVID-19-related health information: a case study in Japan. Humanit Soc Sci Commun. 2022;9(1):270. https://doi.org/10.1057/s41599-022-01293-3. Epub 2022 Aug 15. PMID: 35990766; PMCID: PMC9376898.
153	Changmin Tang	2022	China	Original article	cross-sectional study	Tang C, Chen X, Guan C, Fang P. Attitudes and Response Capacities for Public Health Emergencies of Healthcare Workers in Primary Healthcare Institutions: A Cross-Sectional Investigation Conducted in Wuhan, China, in 2020. International Journal of Environmental Research and Public Health [Internet] 2022;19(19):12204. Available from: http://dx.doi.org/10.3390/ijerph191912204
154	Motilal C. Tayade	2020	India	Letter to Editor	-	Tayade MC. Strategies to tackle by primary care physicians to mental health issues in India in COVId-19 pandemic. Journal of Family Medicine and Primary Care. 2020 Nov 1;9(11):5814-5.
155	Melina K. Taylor	2022	US	Original article	Qualitative	Taylor MK, Kinder K, George J, Bazemore A, Mannie C, Phillips R, Strydom S, Goodyear-Smith F. Multinational primary health care experiences from the initial wave of the COVID-19 pandemic: a qualitative analysis. SSM-Qualitative Research in Health. 2022 Dec 1;2:100041.
156	Kathryn Teng	2022	US	commentary	Qualitative	Teng K, Russo F, Kanuch S, Caron A. Virtual Care Adoption-Challenges and Opportunities From the Lens of Academic Primary Care Practitioners. J Public Health Manag Pract. 2022 Nov-Dec 01;28(6):599-602. https://doi.org/10.1097/PHH.0000000000001548. Epub 2022 Aug 27. PMID: 36037465; PMCID: PMC9555588.
157	Pruthu Thekkur	2022	Sri Lanka	Original article	Mixed-methods	Thekkur P, Fernando M, Nair D, Kumar AMV, Satyanarayana S, Chandraratne N, Chandrasiri A, Attygalle DE, Higashi H, Bandara J, Berger SD, Harries AD. Primary Health Care System Strengthening Project in Sri Lanka: Status and Challenges with Human Resources, Information Systems, Drugs and Laboratory Services. Healthcare [Internet]. 2022 Nov 10;10(11):2251. Available from: http://dx.doi.org/10.3390/healthcare10112251
158	Chris Thomas	2020	UK	Policy brief	-	Thomas C (2020) Resilient health and care: Learning the lessons of Covid-19 in the English NHS, IPPR. http://www.ippr.org/research/publications/resilient-health-and-care
159	Robin N. Thompson	2020	US	Original article	Quantitative: mathematical modelling	Thompson RN et al. 2020 Key questions for modelling COVID-19 exit strategies. Proc. R. Soc. B 287: 20201405. http://dx.doi.org/10.1098/rspb.2020.1405
160	Athanasios Tselebis	2022	Greece	Letter to Editor	-	Tselebis A, Pachi A. Primary Mental Health Care in a New Era. Healthcare (Basel). 2022 Oct 14;10(10):2025. https://doi.org/10.3390/healthcare10102025. PMID: 36292472; PMCID: PMC9601948.
161	Prosper Tumusiime	2022	Congo	Original article	Qualitative: FGD	Tumusiime P, Karamagi H, Titi-Ofei R, Amri M, Seydi ABW, Kipruto H, Droti B, Zombre S, Yoti Z, Zawaira F, Cabore J. Building health system resilience in the context of primary health care revitalization for attainment of UHC: proceedings from the Fifth Health Sector Directors' Policy and Planning Meeting for the WHO African Region. BMC Proc. 2020 Dec 3;14(Suppl 19):16. https://doi.org/10.1186/s12919-020-00203-2. PMID: 33292240; PMCID: PMC7710773.
162	John M	2020	US	Review	Review	Westfall JM, Liaw W, Griswold K, Stange K, Green LA, Phillips R, Bazemore A, Jaén CR, Hughes LS, DeVoe J, Gullett H, Puffer JC, Gotler RS. Uniting Public Health and Primary Care for Healthy Communities in the COVID-19 Era and Beyond. J Am Board Fam Med. 2021 Feb;34(Suppl):S203-S209. https://doi.org/10.3122/jabfm.2021.S1.200458. PMID: 33622839.
163	Wherton J	2022	UK	Original article	mixed methods case study	Wherton J, Greenhalgh T, Hughes G, Shaw SE. The Role of Information Infrastructures in Scaling up Video Consultations During COVID-19: Mixed Methods Case Study Into Opportunity, Disruption, and Exposure. J Med Internet Res. 2022 Nov 10;24(11):e42431. https://doi.org/10.2196/42431. PMID: 36282978; PMCID: PMC9651004.
164	Wilson G	2021	New Zealand	Original article	Qualitative	Wilson G, Windner Z, Dowell A, Toop L, Savage R, Hudson B. Navigating the health system during COVID-19: primary care perspectives on delayed patient care. N Z Med J. 2021 Nov 26;134(1546):17-27. PMID: 34855730.
165	Xu RH	2022	China	Original article	Cross-sectional web-based survey)	Xu RH, Shi LS, Xia Y, Wang D. Associations among eHealth literacy, social support, individual resilience, and emotional status in primary care providers during the outbreak of the SARS-CoV-2 Delta variant. Digit Health. 2022 Mar 25;8:20552076221089789. https://doi.org/10.1177/20552076221089789. PMID: 35355807; PMCID: PMC8958311.
166	Christian Zamiela a	2021	US	Original article	Qualitative	Zamiela C, Hossain NUI, Jaradat R. Enablers of resilience in the healthcare supply chain: A case study of U.S healthcare industry during COVID-19 pandemic. Research in Transportation Economics. 2022 Jun;93:101174. https://doi.org/10.1016/j.retrec.2021.101174. Epub 2021 Dec 24. PMCID: PMC9675944.
167	Ning Zhang	2022	China	Review	Conceptual	Zhang N, Yang S, Jia P. Cultivating Resilience During the COVID-19 Pandemic: A Socioecological Perspective. Annu Rev Psychol. 2022 Jan 4;73:575-598. https://doi.org/10.1146/annurev-psych-030221-031857. Epub 2021 Sep 27. PMID: 34579547.

## Abbreviations

PHC: Primary Health Care
WHO: World Health Organization
SDGs: Sustainable Development Goals
UHC: Universal Health Coverage
PPE: Personal Protective Equipment
GP: General Practitioner
